# Molecular Mechanisms of Epigenetic Regulation, Inflammation, and Cell Death in ADPKD

**DOI:** 10.3389/fmolb.2022.922428

**Published:** 2022-06-29

**Authors:** Ewud Agborbesong, Linda Xiaoyan Li, Lu Li, Xiaogang Li

**Affiliations:** ^1^ Department of Internal Medicine, Mayo Clinic, Rochester, MN, United States; ^2^ Department of Biochemistry and Molecular Biology, Mayo Clinic, Rochester, MN, United States

**Keywords:** ADPKD, epigenetics, post-translational modifications, inflammation, cell death

## Abstract

Autosomal dominant polycystic kidney disease (ADPKD) is a genetic disorder, which is caused by mutations in the *PKD1* and *PKD2* genes, characterizing by progressive growth of multiple cysts in the kidneys, eventually leading to end-stage kidney disease (ESKD) and requiring renal replacement therapy. In addition, studies indicate that disease progression is as a result of a combination of factors. Understanding the molecular mechanisms, therefore, should facilitate the development of precise therapeutic strategies for ADPKD treatment. The roles of epigenetic modulation, interstitial inflammation, and regulated cell death have recently become the focuses in ADPKD. Different epigenetic regulators, and the presence of inflammatory markers detectable even before cyst growth, have been linked to cyst progression. Moreover, the infiltration of inflammatory cells, such as macrophages and T cells, have been associated with cyst growth and deteriorating renal function in humans and PKD animal models. There is evidence supporting a direct role of the PKD gene mutations to the regulation of epigenetic mechanisms and inflammatory response in ADPKD. In addition, the role of regulated cell death, including apoptosis, autophagy and ferroptosis, have been investigated in ADPKD. However, there is no consensus whether cell death promotes or delays cyst growth in ADPKD. It is therefore necessary to develop an interactive picture between PKD gene mutations, the epigenome, inflammation, and cell death to understand why inherited PKD gene mutations in patients may result in the dysregulation of these processes that increase the progression of renal cyst formation.

## 1 Introduction

Autosomal dominant polycystic kidney disease (ADPKD) is the most common genetic disorder of the kidney, caused by mutations in the *PKD1* and *PKD2* genes that encode for transmembrane proteins polycystin-1 (PC1) and polycystin-2 (PC2), respectively (Grantham, 2008). The disease is characterized by the formation and enlargement of fluid filled cysts in the kidneys, and patients with ADPKD eventually develop renal insufficiency and end-stage kidney disease (ESKD), requiring dialysis or kidney transplants (Alam and Perrone, 2010; Grantham et al., 2011). Disease development is associated with hypertension, hematuria, and urinary tract infections (Bajwa et al., 2001; Ecder and Schrier, 2009; Torres and Harris, 2009). In addition, extra-renal complications include cyst formation in other epithelial organs, including the liver and pancreas (Harris et al., 1993). To date, there is only one Food and Drug Administration (FDA) approved treatment, Tolvaptan, for ADPKD (Torres et al., 2012). However, the long-term use of Tolvaptan causes side effects, such as thirst, polyuria, and liver injury (Torres et al., 2012). Therefore, there is an urgent need to develop more effective and safe treatments with the better understanding of molecular mechanisms of ADPKD.

Mutations in the *PKD1* and *PKD2* genes correlate with the development of ADPKD. Individuals with an inherited PKD gene mutation develop detectable renal cysts by 30 years of age (Nicolau et al., 1999). On average, *PKD1* gene mutations lead to ESKD at ∼54 years compared to ∼74 years for *PKD2* (Hateboer et al., 1999). A striking feature of ADPKD is the variability in the phenotype, with the disease severity, the on-set of ESKD, and the spectrum of extra-renal manifestations being highly variable between patients, and even within members of the same family (Milutinovic et al., 1992; Fick et al., 1993; Zerres et al., 1993). Owing to the research focus in our lab over the past 15 years, we will in this review, discuss three molecular mechanisms that may contribute to the disease variability and progression of ADPKD, including epigenetic mechanisms, inflammation, and cell death.

First, epigenetics is broadly defined as a genomic mechanism that reversibly influences gene expression without affecting the DNA sequence (Berger et al., 2009). Epigenetic regulation has been proposed as a potential mechanism to explain disease variability, including ADPKD (Villota-Salazar et al., 2016). We and others found an abnormal upregulation of epigenetic modifiers in kidneys from *Pkd1* animal models and in ADPKD patients (Li, 2011; Bowden et al., 2021). Moreover, inhibition of specific epigenetic factors reduces cyst growth and improves kidney function in preclinical studies, enforcing the role of epigenetic mechanisms in ADPKD (Zhou et al., 2013; Zhou et al., 2014; Zhou et al., 2015; Li et al., 2017c).

Second, we found that the progression of PKD can be influenced by the presence of inflammatory factors such as tumor necrosis factor alpha (TNF-α) (Li et al., 2008) and macrophage migration inhibitory factor (MIF) (Chen et al., 2015) in the cyst fluid. Inhibiting or reducing inflammation by decreasing macrophages for example, has been demonstrated to reduce cyst burden and improve renal function, thereby displaying beneficial effects both on cyst burden and disease progression in preclinical PKD animal models (Swenson-Fields et al., 2013). The upregulation of genes associated with immune and inflammatory responses have also been identified by microarray analysis of ADPKD kidneys (Schieren et al., 2006; Song et al., 2009). Increased T cells (component of the adaptive immunity), specifically localized to cystic lesions, correlate with disease severity. In particular, the role of CD8^+^ T cells in inhibiting ADPKD disease progression has been demonstrated (Kleczko et al., 2018). These studies support the involvement of the inflammatory response, and to a broader scope, the innate and adaptive immune systems in the pathogenesis of ADPKD, and suggest that immunotherapy, such as the reactivation of T cells, might represent a novel therapeutic strategy. Third, we and others have reported that regulatory cell death, including apoptosis (Fan et al., 2013b), ferroptosis (Zhang et al., 2021c) and autophagy (Shillingford et al., 2006), plays a critical role in ADPKD animal models. However, there is a controversy as to whether regulated cell death promotes or delays cyst growth in ADPKD.

In this review, we discuss the roles and molecular mechanisms underlying epigenetics associated with DNA methylation and histone modifiers, inflammation, and programmed cell death in the regulation of disease progression in ADPKD. We debate on the short comings and controversies in the field and how these may impact the discovery of novel mechanisms and treatment options. In addition, we summarize the therapeutic implications and outcomes associated with the therapy of epigenetic, inflammation and cell death. Finally, we provide perspectives on how a better understanding of the diverse mechanisms involved in cyst growth may be applied for combined therapeutic strategies in ADPKD.

## 2 The Roles and Mechanisms of Epigenetic Regulation in ADPKD

Epigenetic alterations which ultimately influence key signaling pathways, have recently been suggested to affect the pathogenesis of ADPKD (Li, 2015). Epigenetic mechanisms including, but not limited to DNA methylation and histone modification, act to regulate accessibility of the DNA by transcription factors to control gene expression (Stuppia et al., 2015). Epigenetic mechanisms play a role in cellular growth and differentiation during development, and as cells mature, these epigenetic modifications change to accommodate the role of the cell. These modifications, including any disease-causing epigenetic changes may be inherited (Robertson, 2005; Greer and Shi, 2012). In addition to regulating the chromatin state, histone modifiers are known to alter gene expression and protein function by post-translational modifications (Miller and Grant, 2013; Sadakierska-Chudy and Filip, 2015). Evidence for alterations in the epigenetic control of gene expression and protein function in ADPKD is accumulating (Li, 2011; Kerr et al., 2019; Li and Li, 2021) and emerging data regarding DNA methylation and histone/lysine modifiers in cystogenesis and ciliogenesis are discussed below.

### 2.1 DNA Methylation and ADPKD

DNA methylation is a stable and heritable epigenetic mark that involves the addition of a methyl group to cytosine residues on the genome by a group of enzymes, named DNA methyl transferases (DNMTs) (Bird, 2002). The DNMT family has four members, DNMT1, DNMT3A, DNMT3B, and DNMT3L. DNMT3A and DNMT3B are referred to as *de novo* methyl transferases, with DNMT3L acting as a stimulator of their catalytic activity (Jeltsch, 2006; Zhang and Xu, 2017). Together, they establish *de novo* methylation patterns which are maintained faithfully during cell replication by DNMT1, hence often referred to as maintenance methyl transferase (Schubeler, 2015). While this modification does not affect the DNA nucleotide sequence, it can modify the availability of the genome to the transcriptional machinery thereby affecting gene expression (Razin and Cedar, 1991). In general, methylation within the gene promoter is typically associated with gene repression and though not well understood, methylation within the gene body is typically associated with sustained or increased gene expression (Saxonov et al., 2006; Shen et al., 2007; Brenet et al., 2011). However, these could deviate from the norm as is the case of ADPKD, as discussed below. DNA methylation has been heavily implicated in human diseases. In cancers, for example, dysregulation of DNA methylation has been reported and inhibitors for DNA methyl transferases (DNMTs), have been developed and approved for the treatment of certain neoplasias, including chronic myelomonocytic leukemia (CMML) and myelodysplastic syndromes (MDS) (Jones et al., 2016). In ADPKD, cysts are believed to arise independently, however, the molecular alterations that underlie cyst formation are poorly understood. Recent studies have identified global methylation patterns of ADPKD patient kidneys and individual cysts, providing evidence of a role for DNA methylation in cystogenesis.

In a pioneering study, the global DNA methylation in ADPKD patient kidneys compared to non-ADPKD kidneys (Woo et al., 2014) was analyzed by methylated-CpG island recovery assay with parallel sequencing (MIRA-seq). This study found that 11,999 genomic fragments, out of the 15 million examined, were differentially hypermethylated, accounting for 91% of all methylation changes. However, only 1,228 genomic fragments were hypomethylated, accounting for 9% of all methylation changes. In addition, this study found that hypermethylation of the *PKD1* gene body (exon 43) in ADPKD patient samples, negatively correlated with the *PKD1* gene expression, suggesting that epigenetic silencing of the *PKD1* gene is involved in kidney cyst development (Woo et al., 2014) (Table 1). As such, Woo et al. theorized that if hypermethylation of the ADPKD genome resulted in cyst growth, then inhibition of the DNMTs could be targeted for therapeutic purposes. In agreement with Woo et al., a second study, utilizing Reduced Representation Bisulfite Sequencing (RRBS) also reported that the *PKD1* gene body was hypermethylated in ADPKD patient kidneys (Table 1). However, contrary to Woo et al., this study found that hypermethylation of the *PKD1* gene body was associated with an increase in *PKD1* gene expression rather than a decrease (Bowden et al., 2018). In addition, unlike Woo et al., this study showed a 2% difference in the methylation status of the genome, with ADPKD patient kidneys being hypomethylated (Bowden et al., 2018). Utilizing methylation-sensitive high-resolution melt (MS-HRM) analysis, a third study demonstrated that hypermethylation of the *PKD1* promoter inversely correlated with gene expression in ADPKD patient blood (Hajirezaei et al., 2020) (Table 1). Fourth, analyzing global methylation patterns of individual cysts derived from the same ADPKD patient (Table 1) revealed that approximately 15% of analyzed fragments exhibited inter-cyst variation in DNA methylation pattern. While the CpG islands and gene body regions demonstrated elevated levels of methylation variation, the intergenic regions had comparatively stable methylation levels within cysts from the same ADPKD patient (Bowden et al., 2020).

**TABLE 1:** Summary of DNA methylation studies in ADPKD.

Sample type	Gene methylation status	Conclusion	Method	Reference
Kidney tissue	*PKD1* gene body hypermethylation	Reduced expression of *PKD1* gene and genes related to cystogenesis in ADPKD.	MIRA-seq	Woo et al. (2014)
Kidney tissue	*MUPCDH* gene promoter hypermethylation	Reduced gene expression and potential novel biomarker	MIRA-seq	Woo et al. (2015)
Kidney tissue	*PKD1* gene body hypermethylation	Differentially hypomethylated fragments of the genome associated with ADPKD.	RRBS	Bowden et al. (2018)
iPSC	No change in DNA methylation pattern in promoters of *PKD1* and *PKD2* genes. DMRs observed between control and PKD mutant iPSCs	Methylation pattern was indicative of PKD-specific epigenetic memory	MeDIP-seq	Kenter et al. (2020)
Kidney cysts	N/A^a^	DMRs in individual cysts matched whole kidney tissue; a subset of loci showed marked DNA methylation heterogeneity	RRBS	Bowden et al. (2020)
Blood	*PKD1* promoter hypermethylation	Inversely correlated with *PKD1* gene expression	MS-HRM	Hajirezaei et al. (2020)

a Coverage was too little for *PKD1* gene methylation analysis.

Abbreviations used: iPSC, induced pluripotent stem cells; DMRs, differentially methylated regions; N/A, none applicable; MIRA-seq, methylated-CpG island recovery assay with parallel sequencing; RRBS, reduced representation bisulfite sequencing; MeDIP-seq, methylated DNA immunoprecipitation sequencing; MS-HRM, methylation-sensitive high-resolution melting.

The potential use of DNMT inhibitors (demethylating agents) for therapeutic purposes in cancers have been acknowledged and well documented. As such, the similarities, and associations between cancer cells and ADPKD suggest that DNMT inhibitors that slow the progress of tumors would have similar effects on cyst growth in ADPKD. At present, the demethylating agents used in clinics are cytotoxic, mutagenic and exhibit lack of specificity towards genes, limiting their clinical application. With the slow progression of ADPKD disease, the long-term use of such drugs may proof harmful. Furthermore, the effect of demethylating agents at pharmacological dosages may depend on the nature and/or extent of the epigenetic changes. ADPKD disease results from different mutations and presents with variable phenotypes, suggesting that the methylation status and subsequent molecular mechanisms may vary. Therefore, understanding epigenetics may help provide new mechanistic insights on cyst development and growth so that broad spectrum and tolerable epigenetic therapy may be developed for ADPKD disease. Advancements in genome-wide technologies have made it possible to analyze genomic methylation levels in ADPKD. Although these studies provide valuable information that point out changes in DNA methylation of the *PKD1* gene, variations have been observed in the methylation status which might be caused by the differences in techniques used for analysis (Table 1). Therefore, use of Whole-Genome Bisulfite Sequencing (WGBS) may be more appropriate to provide a full, unbiased description of the extent of DNA methylation in ADPKD kidneys (Bowden et al., 2021). So far, majority of the methylation analysis studies conducted in ADPKD used kidney tissues from patients. It is important to mention that with the nature of the ADPKD disease (fluid-filled cystic kidneys), it is not practical to obtain kidney biopsies. This suggests that the data presented in the field, arises from kidney tissues obtained at ESKD. We speculate that from the initiation and on-set of cyst growth to ESKD, there may have been changes in the DNA methylation status that are not captured during these analyses. To overcome this problem, blood and/or urine samples from which genomic DNA may be obtained for analysis could be collected from ADPKD patients as the disease progresses. It is our belief that obtaining the DNA methylation status at multiple stages of the disease may provide a more comprehensive epigenetic landscape, which would lay out the foundation for future mechanistic insights and development of therapy in ADPKD. It is important to note that while blood and urine samples are readily accessible, the DNA methylation patterns identified in specific genes obtained from the blood and urine-derived genomic DNA, may not reflect the DNA methylation patterns in the genome of renal cystic epithelial cells and kidneys.

### 2.2 Histone Modifications and ADPKD

In the nucleus, DNA is organized and packaged around histone proteins, which control how accessible the DNA is to the transcription machinery (Cutter and Hayes, 2015). A range of post-translational modifications of these histone proteins (histone “tails”), play a vital role in gene expression. These post-translational modifications determine how tight or loose the histones are packaged, which in turn determines how freely DNA can be transcribed (Shen and Casaccia-Bonnefil, 2008). Several types of histone modifications are known including acetylation, and methylation, phosphorylation, ubiquitination, and sumoylation (Strahl and Allis, 2000; Lunyak and Rosenfeld, 2008; Tan et al., 2011; Graff and Tsai, 2013). Histone modifications at the N-terminal tails on amino acids such as lysine, arginine, serine, threonine, and tyrosine, are catalyzed by specific enzymes that act (Shen and Casaccia-Bonnefil, 2008). Acetylation of histones, catalyzed by histone acetyltransferases (HATs), results in active gene transcription, while deacetylation, catalyzed by histone deacetylases (HDACs) results in reduced levels of gene transcription (Vidali et al., 1988; Kuo and Allis, 1998; Turner, 2000). Histone methylation, regulated by histone methyl transferases (HMTs), results in either the activation or repression of gene transcription, depending on the targeted amino acid residue on the histone tail and/or the number of methyl groups added (mono-, di-, or tri-methylation) (Pedersen and Helin, 2010; Sawan and Herceg, 2010). Removal of methyl groups from the histone tails is catalyzed by histone demethylases (Pedersen and Helin, 2010). In ADPKD, there is accumulating evidence of the dysregulation of enzymes involved in histone acetylation/deacetylation and methylation/demethylation in cystic kidneys (Li, 2011; Li et al., 2017b; Bowden et al., 2021). To the best of our knowledge, there is little to no evidence regarding histone phosphorylation, ubiquitination, and sumoylation in ADPKD. Below, we summarize the role of histone modifying enzymes in ADPKD, with a focus on acetylation and methylation.

#### 2.2.1 Histone Deacetylases in ADPKD

Growing evidence suggest that HDACs are important regulators of PKD genes and/or the signaling pathways that are involved in cystogenesis (Li, 2011). First, it has been proposed that polycystin signaling activates p53, which in turn, in cooperation with HDACs, controls *PKD1* gene expression (Van Bodegom et al., 2006). This study found that the tumor suppressor protein/transcription factor, p53, was a negative regulator of *PKD1*, and that inhibition of HDAC activity rendered the *PKD1* promoter overly sensitive. Second, HDAC5 was identified as a target of the PKD1-dependent fluid stress-sensing in renal epithelial cells (Xia et al., 2010). This study reported that polycystin-1 (PC1) facilitates calcium influx into the cell and subsequent phosphorylation of HDAC5 by protein kinase C. These studies suggested a role for HDACs in the regulation of cystogenesis in ADPKD. Subsequent studies further found that treatment with HDAC inhibitors decreased cyst growth in PKD mutants. In another study, the HDAC class I and II deacetylase inhibitor trichostatin A (TSA), and the Class I HDAC inhibitor, valproic acid, were found to effectively reduce cyst formation, body curvature and laterality in *Pkd2* mutant zebra fish morphants (Cao et al., 2009). Valproic acid also reduced cyst growth in a *Pkd1* mouse model. HDAC6 is upregulated in *Pkd1* mutant mouse cells and was found to activate factors associated with cyst growth, such as EGFR (Liu et al., 2012). Inhibition of HDAC6 with tubacin attenuated cyst growth and improved kidney function through cAMP signaling by preventing Ca^2+^ efflux from the endoplasmic reticulum (Yanda et al., 2017). Additionally, the Class III HDAC SIRT1, was also upregulated in *Pkd1* mutant mouse cells and kidneys to promote cyst growth and treatment with the SIRT1-specific inhibitor, EX-527, reduced cyst growth in *Pkd1* mouse models (Zhou et al., 2013).

**FIGURE 1:**
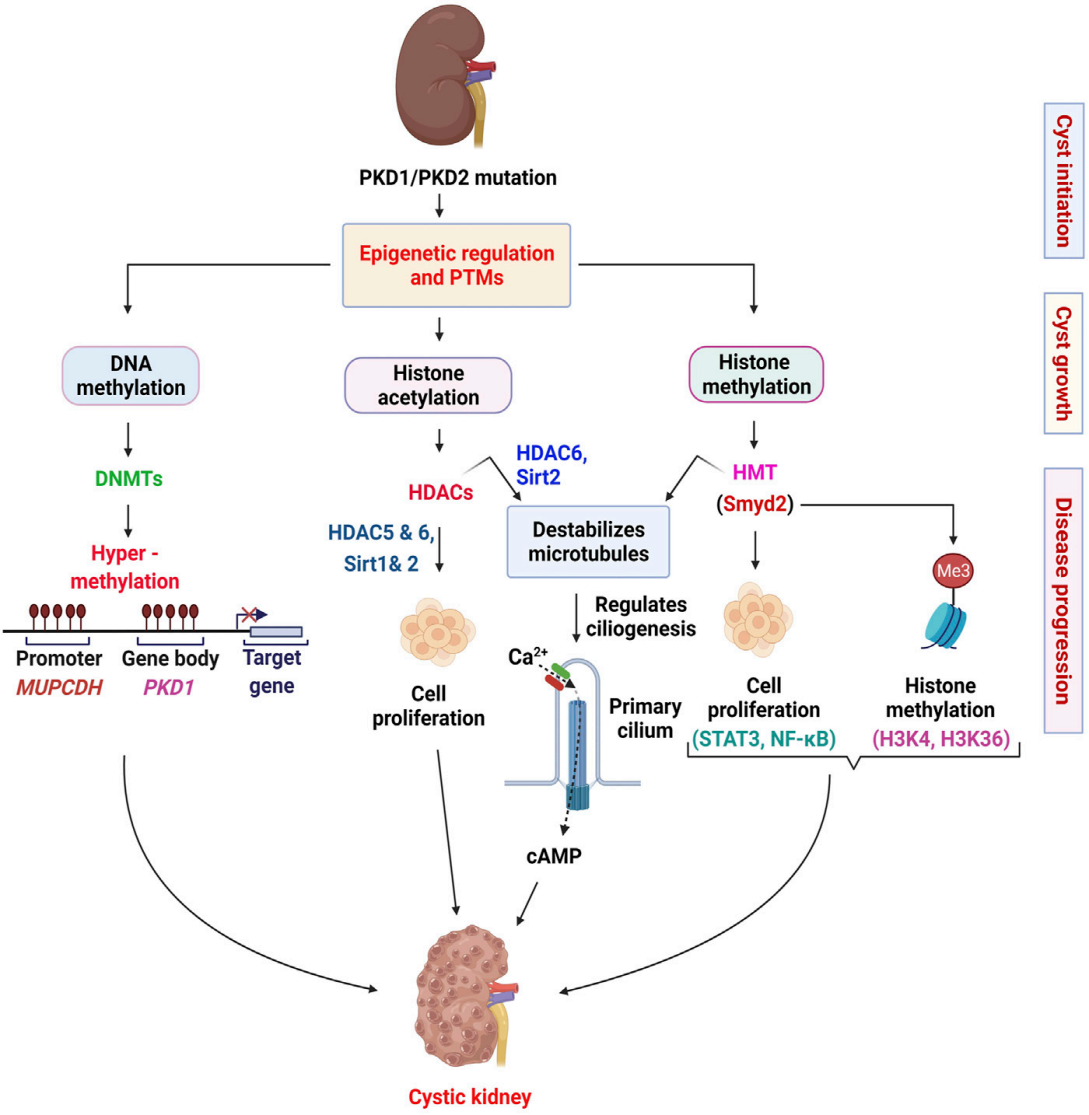
Epigenetic mechanisms implicated in the pathogenesis of ADPKD. In this scheme, we summarize the roles of DNA methyl transferases (DNMTs), histone deacetylases (HDACs), and histone methyl transferases (HMTs) in renal epithelial cells. We indicate the roles of DNMTs in regulating the transcription of *PKD1* and *MUPCDH* genes. In general, we indicate the HDACs and the HMT involved in regulating cell proliferation associated pathways. We also depict the role of HDACs and HMTs in regulating ciliogenesis through deacetylation of α-tubulin (HDAC6 and SIRT2) and methylation of α-tubulin (Smyd2). The involvement of calcium signaling in these processes is possible but uncertain. The various stages of ADPKD (cyst initiation, cyst growth and disease progression) require different epigenetic controls and therefore may require different therapeutic approaches.

The primary cilium is a pivotal organelle for the pathogenesis of cystic kidney diseases, which presents on almost all eukaryotic cells (Eggenschwiler and Anderson, 2007). The cilium is a microtubule-based organelle that functions as a mechano- and chemo-sensor (Nauli et al., 2003). Signaling receptors expressed on the ciliary membrane mediate extracellular sensory signals, creating a response inside the cells (Eggenschwiler and Anderson, 2007; Gerdes et al., 2009; Hildebrandt et al., 2011). Polycystin-1 (PC1) and polycystin-2 (PC2) locate to the cilia and PC2 functions as a Ca^2+^ ion channel (Qian et al., 1997; Yoder et al., 2002; Wang et al., 2019). Furthermore, mutations in cilia-related genes or ablation of cilia result in cystic kidney diseases (Pazour and Rosenbaum, 2002; Yoder et al., 2002). In addition to their role in regulating cell proliferation associated signaling pathways in ADPKD, HDACs also regulate primary cilia structure. HDAC6 is reported to regulate primary cilia disassembly by deacetylating α-tubulin and subsequent studies demonstrated that inhibition of HDAC6 with tubacin, prevents primary cilia resorption (Pugacheva et al., 2007; Zilberman et al., 2009; Ran et al., 2015). The Class III HDAC SIRT2 was also found to regulate primary cilia disassembly and inhibition of SIRT2 by nicotinamide prevented this process (Zhou et al., 2014). Furthermore, inhibition of SIRT2 was found to reduce cyst growth in *Pkd1* mouse kidneys (Zhou et al., 2014). Since HDAC6 and SIRT2 are increased in ADPKD, these studies suggest the involvement of HDAC6 and SIRT2 in the regulation of cystogenesis through cilia-dependent signaling in ADPKD. In sum, these studies suggest that HDACs contribute to ADPKD pathogenesis by regulating both PKD-mediated and cilia-dependent signaling pathways (Figure 1).

#### 2.2.2 Histone Methyl Transferases and ADPKD

Histone methylation commonly occurs in specific arginine and lysine residues at the N- terminal tails of histones (Smith and Denu, 2009). Each arginine methylation can exist in either the mono-methylated, di-methylated symmetrical and asymmetrical methylated states (Byvoet et al., 1972; Bedford and Richard, 2005), whereas each lysine has three possible methylation states: mono-methylated, di-methylated, or tri-methylated states (Murray, 1964; Hempel et al., 1968; Nguyen et al., 2010). Differences in residue methylation and modification states correlate with either gene transcription activation or repression (Zhang and Reinberg, 2001; Li et al., 2007). Lysine methylation at H3 lysine 4 (H3K4) and H3K36 for example are associated with transcriptional activation (Ringrose and Paro, 2004). In contrast, methylation at H3K9 and H3K27 are associated with transcriptional repression (Heard et al., 2001; Schotta et al., 2002; Sims et al., 2003; Ringrose and Paro, 2004). To date, there is limited evidence to support a role for histone methylation in ADPKD pathogenesis. Recently, our group found that the histone/lysine methyl transferase SMYD2, one of the SET and MYND-containing lysine methyl transferases (SMYD), contributed to cyst growth in ADPKD (Li et al., 2017b). SMYD2 can methylate both H3K4 and H3K36 (Brown et al., 2006; Abu-Farha et al., 2008) and non-histone proteins, including p53/TP53 and RB1 (Huang et al., 2006; Cho et al., 2012).

First, we found that SMYD2 expression is increased in *Pkd1* mutant mouse renal epithelial cells and kidneys as well as in ADPKD patient kidneys (Li et al., 2017b). Utilizing *Pkd1* knockout mice and the SMYD2 inhibitor, AZ505, we showed that SMYD2 is a critical mediator of renal cyst growth in ADPKD. In addition, we found that SMYD2 promotes cyst growth in ADPKD via the methylation of H3K4 and H3K36. In particular, SMYD2 regulated cystic epithelial cell proliferation and survival through STAT3 and NF-κB. SMYD2-mediated methylation of STAT3 and NF-κB is important for the activation of these two pathways. We proposed that SMYD2, via two positive feedback loops: SMYD2/STAT3/SMYD2 and SMYD2/NF-κB/SMYD2, promotes cyst development in ADPKD (Figure 1). Second, we determined that SMYD2 is an α-tubulin methyl transferase that together with cyclin-dependent kinases 4 and 6 (CDK4/6), regulates ciliogenesis in renal epithelial cells (Li et al., 2020b). The cross-talk between CDK4/6 and SMYD2 is important for the regulation of ciliogenesis and targeting CDK4/6-SMYD2 signaling affects not only ciliogenesis but also cilia-dependent hedgehog signaling activation in *Pkd1* mutant renal epithelial cells (Li et al., 2020b) (Figure 1). This was the first study to shed light on the contribution of an epigenetic regulator of histone methylation on cyst growth and cilia biogenesis, thereby linking the “tubulin code” (a concept that describes how post-translational modifications that mark subsets of microtubules in the cytoskeleton direct microtubule-based functions) (Park et al., 2016), and cilia-dependent signaling to histone methylation and cyst growth in ADPKD.

The study of epigenetics and associated post-translational modifications have increasingly become an area of interest in ADPKD. The culminative efforts from different research teams have led to the identification of an increasingly complex network of epigenetic mechanisms associated with cystogenesis (Figure 1). Thus far, studies in the field have focused on identifying dysregulated epigenetic modifiers and characterizing their roles and mechanisms in cystogenesis (Zhou et al., 2013; Zhou et al., 2014). Advances were made in the category of histone modifiers, with the identification of the first lysine methyl transferase, Smyd2 and how it is involved in the regulation of cystogenesis and ciliogenesis (Li et al., 2017b; Li et al., 2020b). These studies provided new molecular mechanisms of the disease and provided a novel target for therapeutic purposes. With the advancements made in technology, the field has witnessed an exponential burst in studies aimed at characterizing the epigenome of ADPKD kidneys (Woo et al., 2014; Woo et al., 2015; Bowden et al., 2018; Bowden et al., 2020). These studies have shed light on the role of DNA methylation in the pathogenesis of ADPKD. However, more studies are required to establish a consensus of DNA methylation markers and changes in ADPKD. In addition, how the PKD mutation affects epigenetic mechanisms remains unstudied. Also, the use of epigenetic patterns as markers for cell composition and origin of ADPKD cysts remains unclear. To address these questions, techniques such as whole-genome bisulfite sequencing (WGBS) and single-cell epigenomics sequencing could be applied. Though expensive, the use of WGBS would provide a complete and unbiased description of the extent of DNA methylation in ADPKD kidneys. Single-cell epigenomics sequencing on the other hand would be an effective way to identify the origin, composition, and differentially activated epigenetic mechanisms during the development and progression of ADPKD. Together, these techniques have the potential to identify specific molecular targets that would be more appropriate for ADPKD therapy.

### 2.3 Therapeutic Targets and Therapeutic Implications

The pharmacological control of epigenetic signatures has become a new frontier in different diseases, including cancer. However, the ubiquitous effects of epigenetic changes on pathways limit any potential clinical application in disease treatment. Hypo- and hyper-methylated states in DNA have been associated with ADPKD and thus they represent a potential therapeutic target. DNA methylation is catalyzed by the DNA methyl transferases (DNMTs), and this process potentially contributes to the suppression of gene transcription. This makes it challenging to design drugs whose mechanism of action relies on reactivation of abnormally silenced suppressor genes. There exist multiple classes of DNMT inhibitors (DNMTi) such as nucleoside analog inhibitors azacitidine, and decitabine, however, there is limited information on their efficacy in humans. Thus far, these inhibitors, in combination with chemotherapy have been employed for the treatment of cancers. However, with reports of their cytotoxicity, their use is short-term. With the slow progressive nature of ADPKD pathogenesis, long-term use of DNMTi may result in extensive cytotoxicity.

Changes in histone acetyl groups have also been recognized as epigenetic marks of ADPKD. For histone acetyltransferases (HATs) and histone deacetylases (HDACs), a correlation with cystic burden and severity has been demonstrated in ADPKD mouse models. The activity of HATs may be modulated by bromodomain and extra-terminal motif-containing proteins (BET). In this regard, Zhou et al. demonstrated that targeting the BET bromodomain (BRD) protein, Brd4 with its inhibitor JQ1 (a thieno-triazolo-1,4-diazapine) slows renal cyst growth in *Pkd1* mutant mice (Table 2). With respect to potential modifiers of histone deacetylation, trichostatin A and valproic acid function as HDAC inhibitors (HDACi), and niacinamide acts as Sirtuin (SIRT) inhibitor. Even though HDACi are approved for the treatment of hematological malignancies, their beneficial application in ADPKD is limited to preclinical studies (Table 2). The Sirtuin inhibitor niacinamide on the other hand, is currently undergoing clinical trial for its potential use in the treatment of ADPKD (Table 2).

**TABLE 2:** Summary of clinical trials and preclinical studies targeting epigenetic factors in ADPKD.

Drug	Mediator	Status	Clinical outcome or animal model	Reference
Niacinamide	SIRT1	Phase 2	TKV, eGFR, pain score, urine MCP-1	NCT02558595
Valproic acid	HDAC	Preclinical	*Pkd1* mutant mice	Cao et al. (2009)
JQ1	Brd4	Preclinical	*Pkd1* mutant mice	Zhou et al. (2015)
AZ505	Smyd2	Preclinical	*Pkd1* mutant mice	Li et al. (2017b)

Abbreviations used: TKV, total kidney volume; eGFR, estimated glomerular filtration rate; MCP-1, monocyte chemoattractant protein-1.

Alterations in histone methylation patterns contribute to the epigenetic control of RNA transcription from DNA. Through the transfer of methyl group to lysine or arginine residues, histone methylation, like DNA methylation, is associated with transcriptional repression. However, exceptions exist depending on the methylated residues. Because of the ubiquitous function of the histone-lysine N-methyl transferase enzyme SMYD2, AZ505 may be a promising agent with histone methylation inhibitory properties. However, since SMYD2 plays diverse roles in different cells and organs by regulating distinct substrates, side effects may be unavoidable if SMYD2 inhibitors are used as therapeutic targets in ADPKD.

Despite compelling evidence, the role of epigenetic mechanisms in ADPKD remains unclear. In recent years, research from our lab and others have made substantial contributions towards understanding the mechanisms of epigenetic modifiers in ADPKD disease progression. DNA methyltransferase enzymes and histone modifiers are known to differentially affect the functioning of diverse pathways in cells and organs. One can speculate that oral or intravenous administration of drugs targeting these modifiers may have side effects. Therefore, the use of drug carriers with different affinities for target cells or organs such as kidneys in the case of ADPKD, for the delivery of inhibitors may be a direction of future research.

## 3 The Roles and Mechanisms of Renal Inflammation in ADPKD

The roles of inflammatory response in the pathogenesis of ADPKD has become a central focus in the past decade (Li et al., 2008; Swenson-Fields et al., 2013; Chen et al., 2015; Sadasivam et al., 2019; Zimmerman et al., 2020). ADPKD patients are susceptible to exogenous pathogens due to multi-organ decline caused by loss of renal function, resulting in immune cells proliferation and cytokine secretion. As such, the renal inflammatory response in ADPKD patients has been recognized as a non-initial and secondary effect of cyst progression for a long time (Chen et al., 2015). However, because non-infectious inflammation is present in the early and progressive stages in most *Pkd1* mutant mouse models and ADPKD patients, it is necessary to understand the roles and underlying mechanisms that drive inflammatory response in ADPKD, which may facilitate the development of novel therapeutic strategy for ADPKD treatment. In this section, we discuss how inflammatory response functions in the pathogenesis of ADPKD, and the therapeutic potential associated with these mechanisms.

The two immune systems, innate immune system, and adaptive immune system, which are unique in many aspects, are synergistically mobilized in response to endogenous or exogenous stimulus according to the patterns to recognize the pathogens and response timelines. Innate immune response, also termed non-specific and natural immune response, comes in as the first line of defense against invading pathogens or in response to altered endogenous molecules. This sometimes provides the initiating signal for adaptive immune response, composed of innate immune cells and innate immune associated molecules, including macrophages, dendritic cells, natural killer (NK) cells, natural killer T (NKT) cells, gamma-delta T cells (γδ T cells), eosinophil, neutrophil, mast cells and complement family proteins (Warrington et al., 2011; Netea et al., 2020). The adaptive immune response, also known as acquired or specific immune response, is composed of specialized, systemic cells and processes that eliminate pathogens or prevent their growth through antibodies and cytotoxic T cells. The adaptive immune system relies on the canonical T cells, and B cells by producing antibodies or effector T cells, upon stimulation with antigens presented by antigen-presenting cells (APC) and recognition of T cells receptor (TCR) or B cell receptor (BCR) (Lanzavecchia et al., 1999; Avalos and Ploegh, 2014; Gaudino and Kumar, 2019). NKT and γδ T cells also express TCRs and can also be categorized as adaptive immune cells. Based on their activation patterns, NKT and γδ T cells can also be characterized as innate immune cells. Due to the non-infectious environment during kidney development and the progression of polycystic kidney diseases, the categories of kidney resident immune cells are less complex. According to their origins, all the innate and adaptive immune cells are categorized into three main cell types—granulocytes, mononuclear phagocytes, and lymphocytes. Cells differentiated from granulocytes and mononuclear phagocytes are attributed to innate immune response, while lymphocytes, which include B cells and T cells engage in adaptive immune response. The granulocytes, including eosinophils, neutrophils, and mast cells, are responsible for allergic inflammation. As such, they are detected in peripheral blood and rarely detected in kidneys (Hume, 2008; Malech et al., 2014; Zimmerman et al., 2020). Thus, we will focus on the dominant immune cell types in the kidneys, including macrophages, NK cells, NKT cells, γδ T cells and canonical T cells, to understand their functional roles in the progression of ADPKD.

### 3.1 The Innate Immune Response in the Pathogenesis of ADPKD

#### 3.1.1 The Roles of Macrophages in ADPKD

Kidney macrophages derive from circulating monocytes and resident macrophages. In general, the tissue-resident macrophage population is derived from the yolk sac and fetal liver during development but is complimented by circulating monocytes in response to stimulus (Epelman et al., 2014). Macrophages can be recruited by chemokines, such as monocyte chemoattractant protein-1 (MCP-1), to the damage sites or infectious tissues, switching from monocytes to macrophage. In different organs, macrophages may be attributed unique names, such as Kupffer cells in liver and alveolar macrophage or dust cell in lung (Laskin et al., 2001).

Accumulated evidence exists to support a role for interstitial macrophages in promoting cyst growth in human ADPKD and rodent cystic models (Karihaloo et al., 2011; Swenson-Fields et al., 2013). Treatment with liposomal clodronate to deplete phagocytic cells delayed cyst growth and improved renal function in *Pkd1* ^*fl/fl*^ *:Pkhd1-Cre* mice compared to vehicle treated animals (Karihaloo et al., 2011). Another study reported that M2-like macrophages are abundant in ADPKD patient and mouse kidneys, and depletion of these macrophages led to a milder cystic phenotype and an improved renal function in *Pkd1* mutant mouse kidneys (Swenson-Fields et al., 2013). In general, classical M1-macrophage activation indicates oxidative and pro-apoptotic features, whereas the M2-macrophage activation has proliferative, remodeling and pro-fibrotic effects. This study emphasized a role of M2 macrophages in promoting the progression of ADPKD, highlighting the complexity and differences between M1 and M2 macrophages in this disease.

**FIGURE 2:**
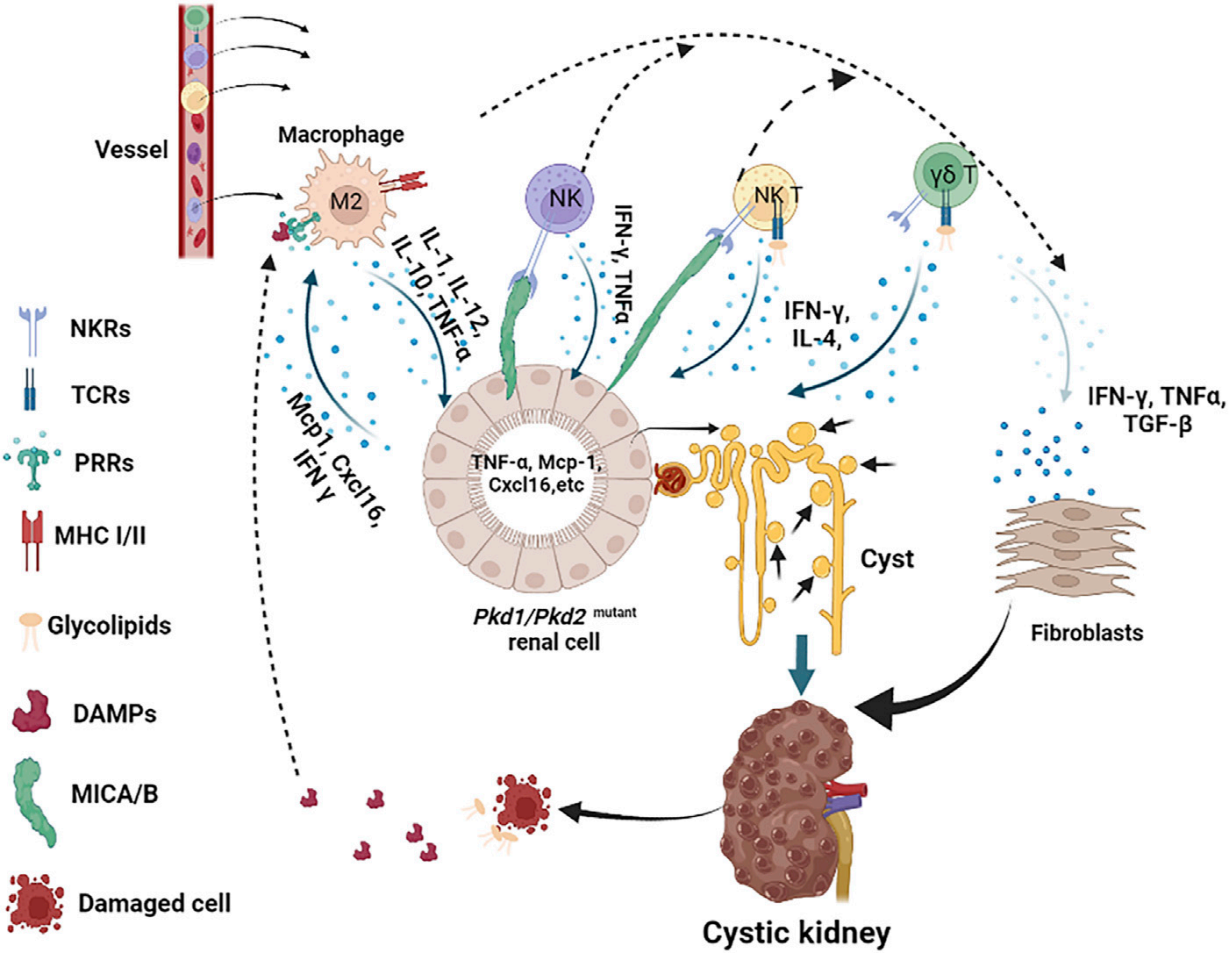
Innate immune response cells and molecules in the pathogenesis of ADPKD. In this scheme, we describe the major innate immune cells in the pathogenesis of ADPKD, including macrophages (referred to M2), NK, NKT and γδ T cells, and the major molecules that participate in this process, including DAMPs, glycolipids, and cytokines. We indicate the main receptors on immune cells, such as MHC I/II on macrophages, TCRs and NKRs on NK, NKT and γδ T cells. *Pkd1* deficient renal cells release Mcp-1 and Cxcl16, which recruit macrophages to the kidney, other cytokines also attract NK, NKT and γδ T cells to infiltrate to kidney as well. Activated macrophages release TNF-α and other cytokines that stimulate renal cell proliferation and induce stress response, resulting in the accumulation of DAMPs in fluid or intestinal or induction of MICA/B on surface of renal cells. Upon recognition of DAMPs and glycolipids released by damaged or dying cells, or MICA/B presented on the surface of cystic cells, activated macrophages, NK, NKT and γδ T cells produce cytokines, such as TNF-α, IFN-ɣ, and TGF-β etc, to further promote cystic renal cell proliferation or renal fibrosis in ADPKD.

The renal interstitial macrophages are mainly recruited and derived from circulating monocytes to kidneys during the disease progression, supporting the hypothesis that chemokines and other factors that attract the infiltration of macrophages should also contribute to ADPKD progression. It has been reported that cultured *Pkd1*-deficient cells express elevated levels of macrophage chemoattractants, including Mcp1 and Cxcl16 (Figure 2), and both of these factors are able to stimulate macrophage migration, suggesting that they may contribute to the recruitment of macrophages to cystic kidneys (Karihaloo et al., 2011). To further understand the mechanisms involved in the recruitment of macrophage in PKD kidneys, we identified the role for the macrophage migration inhibitory factor (MIF) in the recruitment of macrophages to pericystic regions and MIF also regulated other signaling pathways to promote cyst growth in *Pkd1* mutant mice (Chen et al., 2015).

The activation of macrophages is a complicated process, dependent on synergistically coordinated signals from cytokines, ligands, and the corresponding receptors on macrophages (Mosser and Edwards, 2008). In the canonical pathway for macrophage activation and function in response to infection or injury, IFN-ɣ is the most potent macrophage-activating factor and is mainly triggered by viral or parasite infection and released by other immune cells or pathogen affected cells (Muller et al., 2018; Kang et al., 2019; Zhang et al., 2021). Macrophages can also be activated through pattern recognition receptors (PRRs) by an engagement with pathogen-associated molecular patterns (PAMPs) or the damage-associated molecular patterns (DAMPs). PAMPs are presented or released by pathogens, while DAMPs are derived from injured or dying cells (Amarante-Mendes et al., 2018; Roh and Sohn, 2018; Li and Wu, 2021). DAMPs are mainly related to altered self-molecules, including high-mobility group box 1 (HMGB1), S100 proteins, and heat shock proteins (HSPs), etc., reported to be abnormally expressed in diseases, but not or limited in normal situations (Roh and Sohn, 2018). Due to the non-infectious environment of the kidneys during early-stage progression of ADPKD, DAMPs may be the main stimulus for the activation of macrophages compared to PAMPs. The serum levels of HMGB1 are increased in ADPKD patients (Nakamura et al., 2011; Nakamura et al., 2012), while S100A8 and A9 were found to be remarkably increased in both ADPKD patients and mouse models (Lee et al., 2015). Many factors are known to induce the release of DAMPs, including cellular stressors, such as nitric oxide (NO), reactive oxygen species (ROS) and oxidized mitochondrial DNA. Under cellular stress, these factors are released into the cytosol and are responsible for the activation of macrophages (Malysheva et al., 2007; Muralidharan and Mandrekar, 2013; Minton, 2017).

Activated macrophages could either function against inflammatory (M1 macrophage) or further promote inflammatory response (M2 macrophage), respectively, to eliminate infected or injured cells, and assist in the activation of adaptive immune cells, or promote cell proliferation, cell remodeling and fibrosis through different cytokines (Mosser and Edwards, 2008; Arango Duque and Descoteaux, 2014; Krzyszczyk et al., 2018). Activated macrophages can produce cytokines, including IL-1, IL-12, IL-10, TNF-α, etc. (Figure 2). Among those cytokines, TNF-α, which is present in the cystic fluid of human ADPKD kidneys, can disrupt the localization of polycystin-2 to the plasma membrane and primary cilia through a TNF-α induced scaffold protein FIP2, to promote cyst formation in organ cultures and in *Pkd2* mutant mice (Li et al., 2008). The cyst fluid TNF-α may be secreted by activated macrophages or *PKD* mutant cystic renal epithelial cells or both in ADPKD kidneys. These studies suggest that activated macrophage-mediated inflammation plays a role in promoting and/or inducing cystogenesis in the presence of cytokines during cyst expansion. In addition, DAMPs-PRRs activated macrophage may exert its effect through the formation of inflammasomes. Inflammasomes are a group of multimeric protein complexes that consist of a sensor molecule such as PRR, the adaptor protein ASC and caspase 1. Activated inflammasomes play a significant role by releasing IL-1β and IL-18, which are proteolytically activated by caspase 1 (Erlich et al., 2019; Zheng et al., 2020).

#### 3.1.2 The Roles of NK Cells, NKT Cells and γδ T Cells in ADPKD

Natural killer (NK) cells, also called large granular lymphocytes (LGL), are the main innate immune cells that show strong cytolytic function against physiologically stressed cells (tumor and virus-infected cells) and represent 5%–20% of all circulating lymphocytes in humans (Perera Molligoda Arachchige, 2021). NKT and γδ T cells are very similar in many aspects, including: 1) NKT cells and γδ T cells both arise in the thymus, undergo T cell receptor (TCR) gene rearrangement and express CD3 molecule, either CD4 or CD8 molecules, or double negative of CD4/CD8, which is different from conventional T cells (Huang et al., 2014; Krijgsman et al., 2018), and 2) both NKT cells and γδ T cells acquire the expression of the natural killer receptor (NKR) NK1.1 during maturation, including inhibitory NKR (KIRs) and activating NKR (Sawa-Makarska et al.), which is the major difference between NKT cells, γδ T cells and conventional T cells (Krijgsman et al., 2018). Thus, NKT and γδ T cells are at the interface between the innate and adaptive immune system (Dranoff, 2004). The recognition patterns of NKT cells and γδ T cells are more like NK cells rather than conventional T cells, making them pass for innate immune cells instead of adaptive immune cells.

As innate immune cells, NK cells are major effectors of the innate immune system to kill target cells. The role of NK cells in renal fibrosis has been reported, where the accumulation of NK cells in the tubulointerstitial compartment of fibrotic kidneys was correlated with the severity of fibrosis (Law et al., 2017). There is no report regarding the roles of NK cells in the pathogenesis of ADPKD, however, the fact that renal fibrosis is one of the major features of ADPKD, suggests that NK cells may also contribute to renal fibrosis in ADPKD kidneys, and warrants investigation. NKT and γδ T contribute to the main portion of double negative T cell (CD4/CD8 negative, DN), reported to be increased in human ADPKD kidneys compared to controls (Sadasivam et al., 2019). The exact roles of NKT cells in ADPKD are not clear, however, it has been reported that kidney injury induces the activation of NKT cells, and causes hematuria and nephritic casts by damaging glomerular endothelial cells in a perforin-dependent manner through secretion of IFN-γ and other mechanisms and result in kidney dysfunction (Turner et al., 2018). This suggests a role for NKT cells during cyst expansion mediated by kidney injury. Taken together, NK cells, NKT cells and γδ T cells may play a vital role in the progression of ADPKD and need further investigation.

Next, we wanted to address the activation of NK cells, NKT cells and γδ T cells in ADPKD in the absence of pathogens. The fact that NKT cells and γδ T cells harbor two systems of receptors gives them the ability to activated via T cell-like mechanisms or NK cell-like mechanisms. TCRs on NKT and γδ T cells can recognize glycolipids in the context of CD1 family molecules (Liu and Huber, 2011; Pellicci et al., 2020), which is different from conventional T cells, mainly relying on MHC molecules to present peptides antigens. The representative molecule CD1d primarily expressed by antigen-presenting cells (APC), including macrophages, B cells and Dendritic cells, can present both exogenous and endogenous glycolipids in the context of CD1d to activate NKT cells and γδ T cells. Glycolipids include exogenous microbial- and non-microbial-derived glycolipids, and endogenous glycolipids. The latter is mainly released by apoptotic cells or damaged cells or expressed by malignant cells, but rarely detected on normal cells, including gangliosides and sulfatide, phospho-glycerolipids and sphingomyelin (Podbielska et al., 2011; Krijgsman et al., 2018). In ADPKD kidneys, metabolic glycerolipids derived from abnormally proliferative cells or released from DNA damage induced dead cells might be the main glycolipids antigens to stimulate the activation of NKT cells and γδ T cells. In addition to TCRs, NKT cells and γδ T cells also express NK cell receptors. As such, they are activated in a manner comparable to NK cells. Upon stimulation, the outcomes of NK cells, NKT cells and γδ T cells are dependent on the balance between inhibitory and activating signals obtained via the major inhibitory receptors, killer Ig-like receptors (KIRs), and killer cell activating receptors (Sawa-Makarska et al.), respectively (Long et al., 2013). KIRs, provide inhibitory signals upon binding with classical MHC molecules to maintain silence against normal cells (Kumar, 2018). KARs recognize a variety of MHC-like molecules, such as the canonical KAR, NKG2D, which recognizes MHC class I-like molecules A and B (MICA/B) and unique long-binding proteins, which are usually not expressed or lowly expressed in normal cells but robustly expressed on malignant or stress-induced cells, termed as “stress protein” (Long and Rajagopalan, 2002). Thus, these cells play a vital role to maintain homeostasis.

Although the expression of MIC-A/B or other ligands of NKT cells and γδ T cells receptors has not been reported on the surface of cystic cells in ADPKD, the fact that the MICA gene contains an NF-κB-binding site which is necessary and sufficient for transcriptional transactivation of MICA in response to TNFα in primary endothelial cells (ECs) (Lin et al., 2012), suggests a potential of MICA being expressed on cystic renal epithelial cells in ADPKD kidneys. In addition, it has been reported that the regulatory promoter module of MICA/B contains heat shock elements resembling those of HSP70 genes, suggesting that HSP70 and its family proteins also have the potential to stimulate the expression of MICA/B on stress-induced cells (Elsner et al., 2007; Schilling et al., 2015). As an important DAMPs, increased HSP70 also has the potential to induce the activation of NK cells, NKT and γδ T cells through the induction of MICA/B. Loss of self-MHC molecules or abnormal expression of MICA/B in cystic cells induced by HSPs and cyst fluid TNF-α, NK cells, NKT cells and γδ T cells might be activated and produce lots of cytokines. Similarly, upon activation via TCRs, NKT and γδ T cells could also rapidly expand and secrete a range of cytokines, mainly including IFN-ɣ and IL-4 (Coquet et al., 2008; Krijgsman et al., 2018), whereby IFN-ɣ could act as the most potent cytokine to stimulate the activation of macrophages. This would induce a feed-forward loop between these cells in ADPKD kidneys (Figure 2). Taken together, the engagement of receptors of innate immune cells and the potential ligands expressed on cystic cells or released by damaged cells may activate innate immune cells and contribute to the progression of ADPKD (Figure 2).

### 3.2 The Adaptive Immune Response in the Pathogenesis of ADPKD

The adaptive immune response modulated by CD4 or CD8 T cells, and B cells, are responsible for cellular immunity and humoral immunity, respectively. The major difference between innate and adaptive immunity is the specificity of antigen recognition mediated by the TCRs or B cell receptor (BCR). The composition of conventional T cells (TCR αβ), referred to as T cells, are more complicated. T cells including Th1, Th2, CD4/CD25 regulator T cells, and Th17 cells, etc., all belong to CD4 T cells (Zhu et al., 2010). Activated CD8 T cells are mainly cytotoxic T cells, mediating a direct cell killing towards the target cells and releasing of cytokines, such as IFN-ɣ (Bhat et al., 2017; Nicolet et al., 2020). Activated CD4 T cells function significantly different from CD8 T cells, according to the specific phenotype, including the transcription activation or inhibition, and cytokines secretion (Luckheeram et al., 2012). Since the sterile-immune response is the main situation in ADPKD, there is no specific immunogen or pathogen to activate the adaptive immune cells, thus adaptive immune response is more likely a secondary response in ADPKD kidneys.

Reports indicate that both renal CD4^+^ and CD8^+^ T cell numbers are elevated, and correlate with disease severity in the *Pkd1* ^*RC/RC*^ mouse, but with selective activation of CD8^+^ T cells, as analyzed by flow cytometry analysis. In addition, immunodepletion of CD8^+^ T cells worsen ADPKD pathology in one to 3 months *C57Bl/6 Pkd1* ^*RC/RC*^ mice. Furthermore, the expression of T cell recruiting chemokines, CXCL9/CXCL10, which were secreted by cystic epithelial cells and renal interstitial cells, were significantly increased in kidneys of *Pkd1* ^*RC/RC*^ mice compared to those in kidneys from wildtype mice (Kleczko et al., 2018). These results suggested a protective role of CD8^+^ T cell in ADPKD and implied that the increase in T cells was as a result of extrarenal recruitment rather than the amplification of resident T cells. Besides CD8 T cells, there is no report defining the roles of CD4 T cells in ADPKD. A study found the CD4 T regulatory cells worsen chronic kidney disease (CKD) or end-stage kidney disease (ESKD) (Hartzell et al., 2020), suggested that CD4 T regulatory cells might also promote the progression of ADPKD.

Studies have reported increases in B cell numbers in ADPKD, however, little is known on the roles of B cells in disease progression. Furthermore, the mechanisms of activation of adaptive immune cells are still largely unknown. It has been proposed that DNA damage response mediated ubiquitous cell proliferation across the cystic kidneys, is the cause of the increases in T and B cells in ADPKD kidneys. Reports indicate that loss of polycystin-1 (PC1) impairs DNA damage response and induces cell proliferation of PC1 deficient cells in the kidneys (Zhang et al., 2021b). Because PC1 and PC2 are also expressed in lymphocytes, the intrinsic rate of DNA damage and the susceptibility to DNA damage agents are also increased in the peripheral blood lymphocytes from ADPKD patients (Aguiari et al., 2004; Li et al., 2013). Thus, PC1 deficiency may also induce cell proliferation in lymphocytes in an immune-recognition independent manner. In addition, activated innate immune cells could produce cytokines, such as IFN-ɣ, TNF-α TGF-β and IL-1β to regulate the amplification of adaptive cells and adaptive immune response, especially T cells. For example, by activating inflammasome, innate immune cells including macrophage, produce IL-1β, which is a pro-inflammatory cytokine that stimulates T cell activation, resulting in T cell differentiation under different conditions (Wan and Flavell, 2007; Croft, 2009; Van Den Eeckhout et al., 2020).

### 3.3 Prospective in Immune Therapy in the Treatment of ADPKD

Immunotherapies to restore the dysregulated immune response in ADPKD by either the inactivation of the overactivated cell types or the activation of the protective cell types would benefit the patients. Several immunotherapeutic strategies have been evaluated in *Pkd1* mutant mouse models (Table 3).

**TABLE 3:** Prospective immunotherapy strategies in the treatment of ADPKD.

Category	Targets	Methods and outcomes	References
Targeting abnormal immune cells	CD8^+^ T cells	Immunodepletion of CD8 T cells worsens ADPKD phenotype	Kleczko et al. (2018)
	CD4^+^ Treg cells	Antibody against CD25 attenuate the progression of ADPKD.	Onda et al. (2019)
	Macrophages	Exhaustion of macrophages delayed cyst growth	Swenson-Fields et al. (2013)
	Macrophages	Genetic deletion of MIF delayed cyst growth	Chen et al. (2015)
	NK, NKT and γδ Τ cells	Neutralization with antibodies against KAR NKG2D prevents the activation of NKG2D-expressing cells	Lodoen et al. (2003), Steigerwald et al. (2009)
Targeting cytokine secretion	IFN-γ	Neutralization of IFN-γ with antibodies inhibits the proliferation	Prencipe et al. (2018)
	TNF-α	Neutralization of TNF-α might overactivation of inflammation in ADPKD.	Li et al. (2008)
Targeting cytokine regulators	Caspase 1	Targeting caspase 1 with its inhibitor could suppress the inflammasome activation and reduce IL-1β and IL-18	Flores et al. (2020), Liang et al. (2020)
	NLRP3	Targeting NLRP3 with its inhibitor could reduce IL-1β and IL-18	Zahid et al. (2019)
Targeting the regulatory machinery of immune response	CBP/p300 coactivators, KMTsetc.	CBP/p300 coactivators regulates the transcription of TNF-α and its family members	Falvo et al. (2000), Granja et al. (2006)
	Set7	Stress-mediated induction of histone methyltransferase Set7, leads to promoter modification on MCP-1 through Set7-mediated H3K4 methylation	Batista and Helguero (2018)

(1) Targeting abnormal immune cells: First, it was reported that immunodepletion of CD8 T cells worsens ADPKD phenotype, suggesting that activation of CD8 T cells should attenuate the progression of ADPKD. Second, it has been found that CD4 Treg cells promote cyst growth, suggesting that specifically targeting CD4 Treg cells with antibody against CD25 rather than CD4 to delete Treg cells might be better than targeting other subsets of CD4 T cells, since CD25 was the first surface marker used to identify Tregs (Onda et al., 2019). Third, it has been confirmed that induced exhaustion of macrophages or genetic deletion of MIF delayed cyst growth and improved renal function (Swenson-Fields et al., 2013; Chen et al., 2015), supporting the hypothesis that targeting macrophages and factors associated with macrophage recruitment and function is a potential strategy for the treatment of ADPKD. With regards to other immune cell types such as NK, NKT and γδ T cells, the neutralization with antibodies against activating receptors may have beneficial effect. One possibility is to target KAR NKG2D with an antibody, which can prevent the activation of NKG2D-expressing cells (here referring to NK, NKT and γδ T cells) and cytokine secretion by inducing rapid internalization of antigen-antibody complex upon binding to NKG2D (Lodoen et al., 2003; Steigerwald et al., 2009).

(2) Targeting cytokine secretion: It has been reported that neutralization of IFN-γ with antibodies inhibits the proliferation and activation of immune cells in virus-infection model (Prencipe et al., 2018), and may be a strategy to be tested in ADPKD mouse models and patients. The neutralization of TNF-α might also provide another possibility to reduce overactivation of inflammation in ADPKD (Li et al., 2008).

(3) Targeting cytokine regulators: Caspase 1 is responsible for the release of both IL-1β and IL-18. Targeting caspase 1 with its inhibitor could suppress the inflammasome activation and downstream effects, which has already been tested in Alzheimer’s disease models and ischemia-associated blood-brain barrier dysfunction (Flores et al., 2020; Liang et al., 2020). In addition to the regulation of caspase 1, inhibitors targeting NLRP3, another component of inflammasome, have been evaluated in other disease associated cell models (Zahid et al., 2019).

(4) Targeting the regulatory machinery of immune response: As discussed above, epigenetic mechanisms contribute to ADPKD progression. Epigenetic regulation plays a role in renal inflammation in kidney diseases by regulating the expression of cytokines through chromatin modifications at the transcriptional levels. For example, the transcription of TNF-α and its family members is associated with several epigenetic regulators responsible for histone acetylation and methylation, including the CBP/p300 coactivators (Falvo et al., 2000; Granja et al., 2006). It has also been reported that stress-mediated induction of histone methyltransferase Set7, leads to promoter modification on MCP-1 through Set7-mediated H3K4 methylation (Batista and Helguero, 2018). These studies suggest that epigenetic regulation should also be involved in renal inflammation in PKD, providing an alternative strategy for the combination of immuno- and epigenetic therapy in ADPKD treatment.

In summary, both innate and adaptive immune responses participate in the pathogenesis of ADPKD through unique mechanisms. The roles of macrophages have been extensively investigated in PKD in the past decade. The roles of T cells in PKD have also been examined. So far there is not any clinical immunotherapeutic strategy for the treatment of ADPKD, all these immunotherapeutic strategies listed above are prospective immunotherapy according to the current studies in this field. (Table 3). Next, we may focus on the roles and mechanism of NK, NKT and γδ T cells in the regulation of PKD progression. In addition, the interaction of innate immune system and adaptive immune system should also be determined. Furthermore, the effect of immunotherapy should be assessed in pre-clinical and clinical trials. Overall, synergistical network of innate and adaptive immune cells, cytokines, immune related components, as well as the upstream regulatory factors and downstream effectors, make inflammation an important process in the pathogenesis of ADPKD. Epigenetic mechanisms contribute to the regulation of inflammatory response, and eventually regulate cell proliferation and cell death. In turn, the cytokines and other inflammatory factors regulate the expression and activity of these epigenetic factors, and regulate cell fates as well, such as proliferation and death. Thus, the integration of inflammation, epigenetic regulation and cell death should be investigated, in order to identify useful mechanisms to develop novel therapeutic strategies for the treatment of ADPKD in the future.

## 4 The Roles and Mechanisms of Regulated Cell Death in ADPKD

Cell death is an essential process to maintain homeostasis in the human body and is involved in diverse physiological processes including embryonic development and elimination of harmful or unnecessary cells (Norbury and Hickson, 2001; Fuchs and Steller, 2011; Tower, 2015). There are distinct types of regulated cell death, including apoptosis, autophagy, necrosis, and the most recently identified ferroptosis (Table 4). Thus far, three types of cell deaths, including apoptosis, autophagy and ferroptosis have been associated with the pathogenesis of ADPKD, and accumulated evidence suggests that targeting cell death pathways may be a potential therapeutic strategy for ADPKD treatment (Capuano et al., 2022).

**TABLE 4:** Diverse types of cell death in ADPKD.

Cell death type	Basic features	Biochemical features	Morphological features	Detection methods
Apoptosis	Type1 programmed cell death	Activation of caspases, oligonucleosomal DNA fragmentation	Plasma membrane blebbing, nuclear condensation and fragmentation, apoptotic bodies	TUNEL, DNA ladder, DNA content analysis, apoptosis enzyme-linked immunoassay, annexin binding assay, LDH activity assay, mitochondrial membrane potential assay
	Reversible			
Autophagy	Type2 programmed cell death	Increased lysosomal activity, LC3I to LC3II transformation	Formation of double-membraned autolysosomes	Western blotting or Fluorescence Microscopy of LC3 (marker protein for autophagosomes) and p62 (autophagy substrate)
	Reversible			
Ferroptosis	Reversible	Iron and ROS accumulation, inhibition of xCT and reduced GSH.	Increased density of outer cell membrane, ruptured outer mitochondrial membrane	Iron assay kit, GSSG/GSH Quantification kit, Glutamine assay kit

Abbreviations used: ROS, reactive oxygen species; xCT, light-chain subunit of SLC7A11 (system xc− cystine/glutamate antiporter); GSH, glutathione; GSSG, oxidized glutathione; LDH, lactate dehydrogenase.

### 4.1 The Mechanisms of Regulated Cell Death

#### 4.1.1 The Mechanism of Apoptosis

**FIGURE 3:**
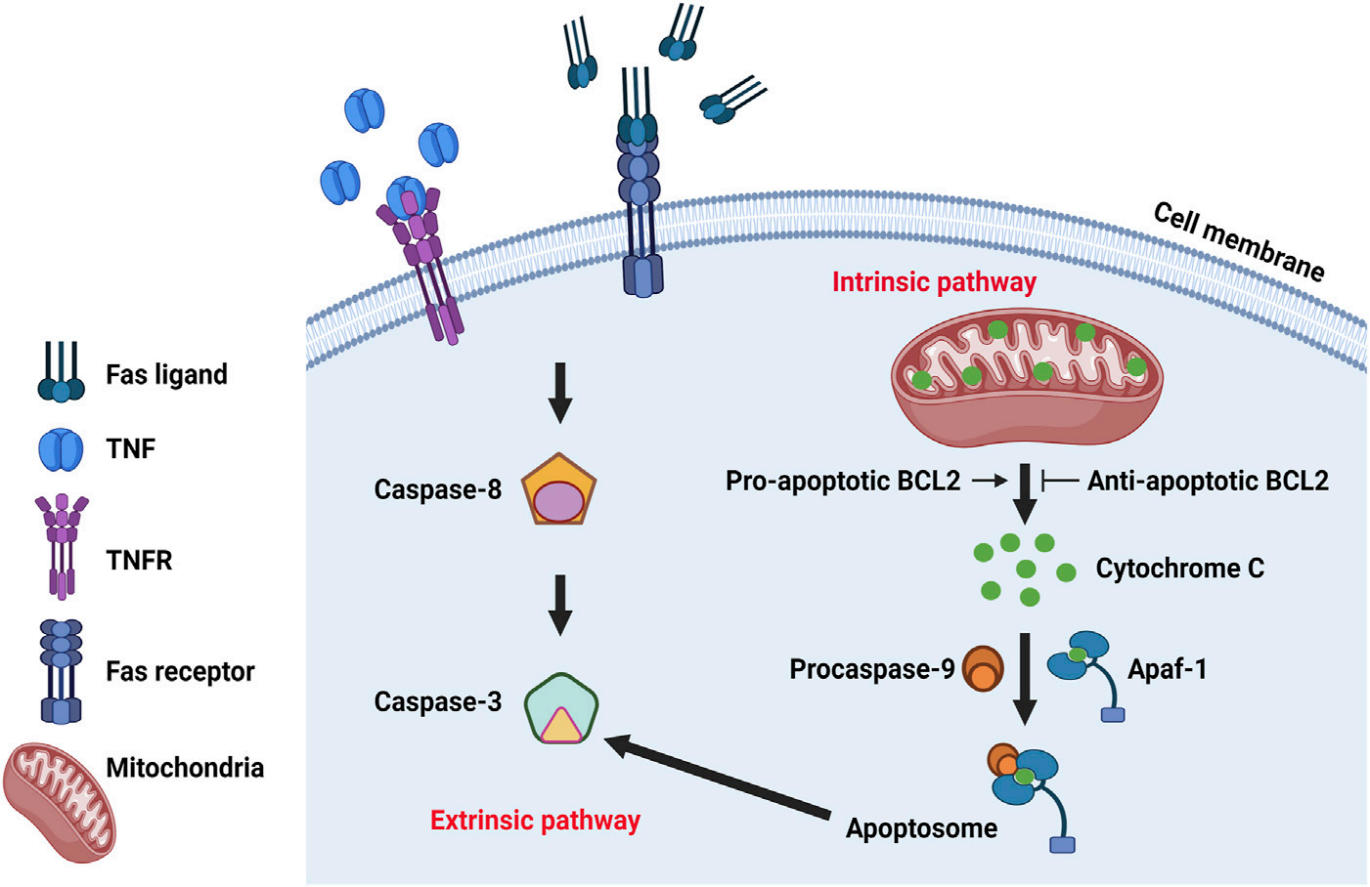
The molecular mechanism of apoptosis. Classic regulatory pathways of apoptosis include extrinsic pathway and intrinsic pathway. Intrinsic pathway is mitochondria dependent and regulated by BCL2 protein family which can influence the release of cytochrome C from the mitochondria. Cytochrome c can bind to the cytosolic protein Apaf-1 and promote the formation of an apoptosome to recruit and activate caspase-9, which in turn activates caspase-3 and leads to cell apoptosis. Extrinsic pathway is initiated by the transmission of death signals from the cell’s surface through the binding of ligands and the death receptors, resulting in the aggregation and recruitment of initiator caspases that subsequently activates executioner caspase 3, leading to apoptosis.

Apoptosis is the process of programmed cell death characterized by membrane shrinkage, chromatin condensation, nuclear fragmentation (pyknosis) and eventually the formation of apoptotic bodies (Elmore, 2007; Obeng, 2021). Apoptosis functions at all stages of human life, including embryonic development and aging to eliminate unwanted cells, and as a defense mechanism to remove injured cells that have been damaged beyond repair (Ke et al., 2018). There are two main pathways in apoptosis, the intrinsic and the extrinsic pathways (Figure 3), which are initiated and executed by two groups of caspases (cysteine-aspartic proteases), including the initiator caspases (caspases 2, 8, 9, 10) and the executioner caspases (caspases 3, 6, 7) (Zhou and Li, 2015). Caspases are a family of protease enzymes which can cleave their substrates at aspartic acid residues (Alnemri et al., 1996; Julien and Wells, 2017). The activation by initiator caspases can be induced by external death ligands or the release of cytochrome c from mitochondria, which initiate the apoptotic signals and directly cleave and activate executioner caspases for the execution of apoptotic program (Duclos et al., 2017).

The intrinsic pathway is mitochondria-dependent, initiated when an injury occurs within the cell and mainly regulated by proteins of the BCL2 (B-cell lymphoma 2) family, which are evolutionarily conserved with shared Bcl-2 homology domains, including BH1, BH2, BH3, and BH4 (Boletta et al., 2000). BCL2 family proteins can be divided into three types: pro-apoptosis (Bcl-2, Bcl-xL, etc.), anti-apoptosis (BAX, BAK, etc.) and regulatory (BAD, BIK, BIM, etc.) members (Reed, 1998; Kale et al., 2018). Diverse types of BCL2 family contain different BH domains. Anti-apoptotic proteins usually contain BH1 and BH2 domains and pro-apoptotic proteins usually contain a BH3 domain which is essential for dimerization with other proteins of the Bcl-2 family and crucial for their killing activity. Some pro-apoptotic proteins also contain BH1 and BH2 domains (Bax and Bak). The BH3 domain may also be present in some anti-apoptotic proteins, such as Bcl-2 or Bcl-x(L) (Kale et al., 2018). The balance between pro- and anti-apoptotic Bcl-2 family members is essential to control the activity of caspases (Swanton et al., 1999; Marsden et al., 2002; Hatok and Racay, 2016; Roufayel, 2016). BCL2 family proteins are found in the mitochondrial membrane, where the pro-apoptotic proteins can promote the release of cytochrome c from mitochondria while anti-apoptotic proteins can inhibit its release (Adams and Cory, 1998; Korsmeyer, 1999; Morales-Cruz et al., 2014; Pena-Blanco and Garcia-Saez, 2018). Cytochrome c can bind to the cytosolic protein Apaf-1 and promote the formation of an apoptosome to recruit and activate caspase-9, which in turn can activate caspase-3 and leads to cell apoptosis (Bratton and Salvesen, 2010; Elena-Real et al., 2018).

The extrinsic pathway which begins outside the cell, is initiated by the transmission of death signals from the cell’s surface to intracellular signaling pathways through the binding of specific death receptors to their ligands. This results in the aggregation and recruitment of initiator caspases that subsequently activates executioner caspase 3, leading to apoptosis (Elmore, 2007; Green and Llambi, 2015). Several death ligands and their corresponding death receptors have been identified, including Fas ligand and Fas receptor (FasL/FasR), tumor necrosis factor (TNF) and its receptor 1 (TNF-α/TNFR1), Apo3L/DR3, Apo2L/DR4 and Apol2L/DR5 (Walczak, 2013). TNFRs are death receptors that belong to members of the TNF receptor superfamily with shared cysteine-rich extracellular domains and a cytoplasmic death domain (Micheau and Tschopp, 2003; Dostert et al., 2019). The classical extrinsic pathway is mediated by FasL/FasR and TNF-α/TNFR1 (Nagata, 1999; Rath and Aggarwal, 1999; Yanumula and Cusick, 2022). The binding of FasL with FasR results in the recruitment of the adaptor protein FADD (Fas-associated death domain) (Wajant, 2002; Caulfield and Lathem, 2014) which then associates with procaspase-8 to form the death-inducing signaling complex (DISC), leading to the activation of procaspase-8 (Kischkel et al., 1995; Wajant, 2002). Conversely, TNF binds to TNFR resulting in the transient recruitment of TRADD (TNF-related apoptosis inducing ligand), TNF receptor-associated factor 2 (TRAF2), TRAF5, cellular inhibitor of apoptosis 1 and 2 (cIAP1/2) and receptor interacting protein 1 (RIP1) to form pro-survival complex I (Micheau and Tschopp, 2003; Shi and Sun, 2018) which can activate nuclear factor κB (NF-κB) and JNK pathways to regulate the expression of pro-survival genes, including the cellular FLICE-like inhibitory protein (cFLIP) (Liu et al., 1996; Dhanasekaran and Reddy, 2008). With the deubiquitination of RIP1, TRADD and RIP1 become disassociated from complex I, then RIP1 associates with FADD and caspase-8 to form complex II, the so-called death complex to trigger cell death (Amin et al., 2018).

#### 4.1.2 The Mechanism of Autophagy

Autophagic cell death is another form of programmed cell death characterized by the large-scale accumulation of vacuolated-like structures called autophagosomes (Kroemer and Levine, 2008; Garg et al., 2015). Autophagy is an important physiological process that has been associated with ADPKD pathology and is regulated by known PKD associated signaling pathways. For example, the mammalian target of rapamycin (mTOR) inhibits autophagy, while the AMP-activated protein kinase (AMPK) is known to activate autophagy.

Autophagy process includes five steps: initiation, elongation, maturation, fusion, and degradation. The activation and phosphorylation of Unc-51 like autophagy activating kinase (ULK1) protein complex, inhibited by the mTOR pathway (including mTORC1 and mTORC2) and activated by the AMPK pathway, initiates autophagy. Under normal conditions, mTORC1 inhibits the activation of the ULK1 complex by phosphorylating Ser 757 of ULK1 and interrupting the interaction between ULK1 and AMPK. Upon stimulation by cellular or environmental stresses, inhibition of mTORC1 results in the dephosphorylation of ULK1. Meanwhile, AMPK directly activates ULK1 through phosphorylation of Ser 317 and Ser 777 in ULK1 (Kim et al., 2011). Phosphorylation of ULK1 initiates the process of autophagy as described above. Activated ULK1 then phosphorylates other components of the ULK1 complex (including FIP200, ATG13 and ATG101), and recruits the PI3KC3 complex (including BECN1, Vps15, Vps34, NRBF2, AMBRA1, Atg14) to coordinate the nucleation and biogenesis of autophagosome (Meijer and Codogno, 2006; Mizushima, 2010; Lin and Hurley, 2016). ATG9 is the only transmembrane protein in the ATG protein family that functions as a membrane carrier to deliver lipids to the forming autophagosome from several cellular membranes, including the plasma membrane, mitochondria, recycling endosomes and Golgi complex (Matoba et al., 2020; Sawa-Makarska et al., 2020). The process of expansion and maturation of the autophagosome membrane involves two ubiquitin-like conjugation systems: the conjugation of ATG12 to ATG5, and the conversion of LC3 I to LC3 II (Mizushima, 2020). The conjugation of Atg12 to Atg5 occurs at Lys130 through the activation of E1 enzyme Atg7 and the E2-like Atg10 (Mizushima et al., 1998; Otomo et al., 2013). The Atg12-Atg5 conjugate then forms a large protein complex with Atg16, acts as the E3 ligase for the conjugation of LC3 I to PE (phosphatidylethanolamine). Atg4 cleaves the C-terminal arginine of pro-LC3 to form LC3 I. The conversion of LC3 I to LC3 II is also a ubiquitin-like conjugation reaction. E1, E2, and E3-like enzymes are Atg7, Atg3, and Atg12-Atg5-Atg16 (Tanida et al., 2004). LC3 II is a characteristic marker of autophagic membranes and can recruit selective cargo to the autophagosome via its interaction with cargo receptors (Kabeya et al., 2000; Tanida et al., 2004). The maturation of the autophagosome leads to the autophagosome-lysosome fusion. The lysosome is a double-membrane cell organelle that contains digestive enzymes. The enzymes contained in the lysosome ensure the degradation of the cargo and cargo receptors which are recycled to be used again during cellular metabolism (Kriegenburg et al., 2018).

#### 4.1.3 The Mechanism of Ferroptosis

**FIGURE 4:**
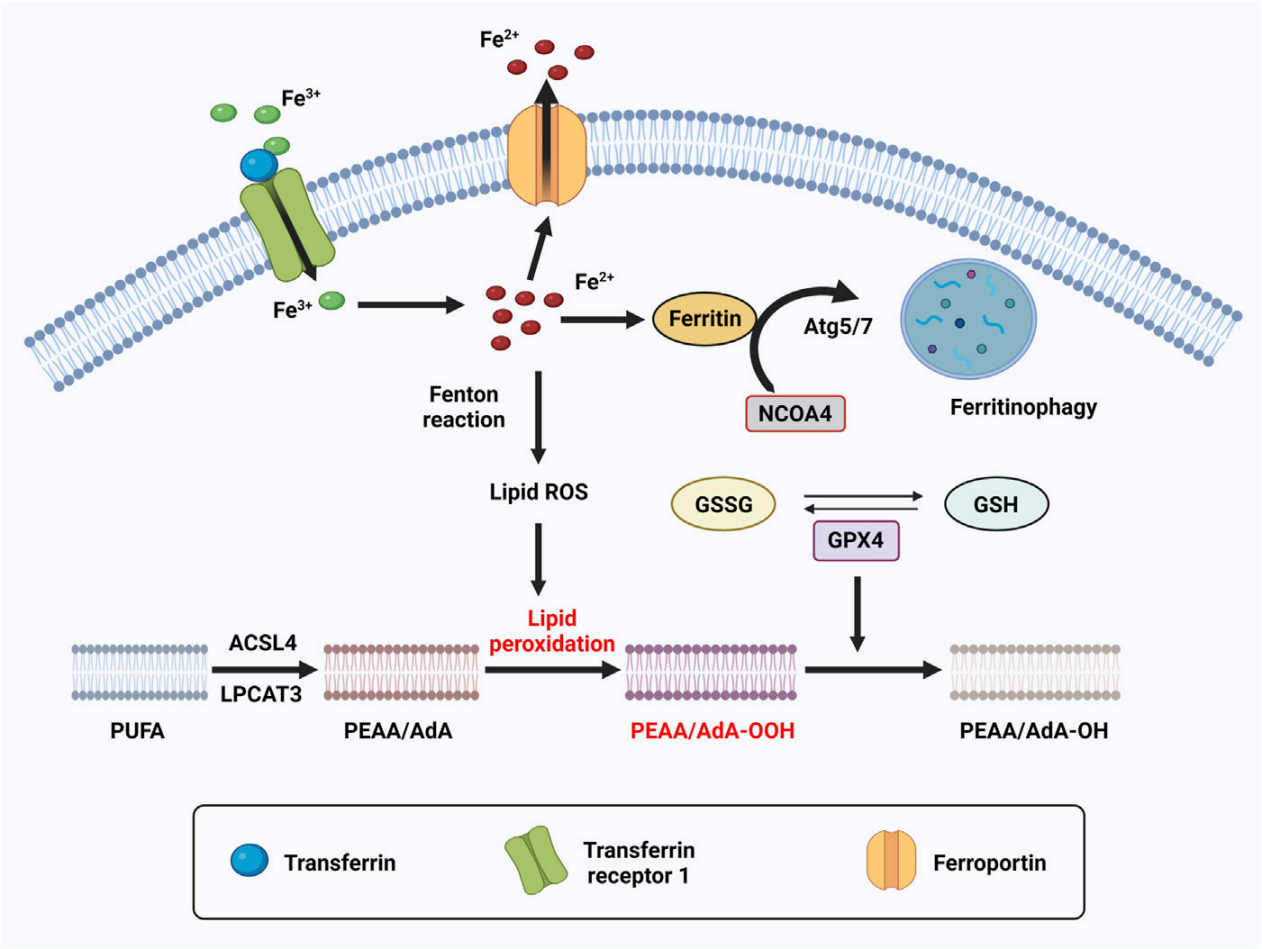
The molecular mechanism of ferroptosis. Ferroptosis is an iron dependent form of cell death. The metabolism of iron includes iron uptake (transferrin receptor), iron export (ferroportin), iron storing (ferritin), and ferritinophagy. Ferritinophagy is a selective autophagy of ferritin which mediated by Atg5/7 and NCOA4. Excess can directly induce ferroptosis through lipid peroxidation. GPX4, converts PEAA/AdA-OOH to PEAA/AdA-OH and inhibits ferroptosis. This reaction occurs through the use of glutathione (GSH) as a substrate.

Ferroptosis is an iron dependent form of cell death which can be induced by small molecules such as erastin and Ras-selective lethal small molecule 3 (RSL3) (Dolma et al., 2003; Yang and Stockwell, 2008), and can be inhibited by specific inhibitors such as ferrostatin-1 (Fer-1), liproxstatin-1 and vitamin E. In addition, iron chelators and lipophilic antioxidants can prevent ferroptosis (Xie et al., 2016). The failure of glutathione-dependent antioxidant defenses initiates ferroptosis, leading to uncontrolled lipid peroxidation and eventually cell death (Li et al., 2020a; Zhang and Li, 2022). Ferroptosis is regulated by Glutathione/GPX4 (glutathione peroxidase 4) signaling pathways, iron metabolic signaling pathway and lipid metabolic signaling pathway as indicated in Figure 4 (Figure 4) (Li et al., 2020a). GPX4 is a selenoenzyme which can convert GSH (glutathione) into oxidized glutathione (GSSG) and reduces membrane cytotoxic lipid hydroperoxides to maintain cellular redox homeostasis and prevent the iron (Fe^2+^)-dependent formation of toxic lipid reactive oxygen species (ROS) (Seiler et al., 2008). Inhibition of GPX4 can lead to the accumulation of lipid peroxides and induction of ferroptosis (Yang and Stockwell, 2008; Yang et al., 2014).

Iron is one of the most abundant transition metals and is an essential element for many living organisms. Because iron-dependent oxidative damage is characteristic for ferroptosis, iron metabolism is controlled by ferroptosis in many aspects. The metabolism of iron includes iron uptake (transferrin receptor), iron export (ferroportin), iron storing (ferritin), and ferritinophagy, selective autophagy of ferritin mediated by lysosome and NCOA4 (nuclear receptor coactivator 4). Excess heme and non-heme iron can directly induce ferroptosis (Li Q. et al., 2017), and both heme and non-heme iron-containing enzymes, such as ALOXs, NOXs, and CYP can promote lipid peroxidation in ferroptosis. During ferroptosis, a phenomenon in which increased labile iron is released by the cell is referred to as ferritinophagy. NCOA4 is a selective cargo receptor which functions in ferritinophagy. The genetic inhibition of NCOA4 inhibited ferritin degradation and suppressed ferroptosis. In contrast, overexpression of NCOA4 increased ferritin degradation and promoted ferroptosis (Hou et al., 2016). Iron chelators are drugs that can remove extra iron from the body, blocking ferroptotic cell death both *in vitro* and *in vivo*. Ferritin is regulated by ATG5-ATG7 and NCOA4 pathways, as well as IREB2 (Iron Responsive Element Binding Protein 2). In addition, p62-Keap1-NRF2 and HSPB1 (Heat Shock Protein Family B Member 1) signaling pathways can also regulate iron metabolism.

Lipid peroxidation is a process of oxidative lipid degradation that eventually leads to ferroptosis. During this process, ROS such as oxygen free radicals attack lipids, especially polyunsaturated fatty acids (PUFAs). PUFAs are preferentially incorporated into phospholipids such as phosphatidylethanolamines (PEs) and transformed into PUFA-PEs by two enzymes, ACSL4 (Acyl-CoA synthetase long-chain family member 4) for synthesizing PEs and LPCAT3 (lysophosphatidylcholine acyltransferase 3) for lipid remodeling. Therefore, blocking the expression of ACSL4 and LPCAT3 results in the suppression of esterification of PEs reducing the accumulation of lipid peroxide substrates in cells, thus inhibiting ferroptosis (Kagan et al., 2017).

### 4.2 The Roles of Regulated Cell Death in ADPKD

#### 4.2.1 Apoptosis in ADPKD

Aberrant apoptosis and apoptotic pathways were first detected in human ADPKD, the congenital mouse model of ARPKD (*cpk*), and the *pcy* mice (Woo, 1995). In the past decades, an increasing number of studies have demonstrated that apoptosis plays an essential role in the regulation of cystogenesis in ADPKD. However, whether apoptosis is increased or decreased in ADPKD kidneys, and whether induction of apoptotic cell death promotes, or delays cyst growth remains controversial.

First, studies found that apoptosis is elevated in rodent ADPKD models. In the *Han:SPRD-Cy (Cy)* rat model which closely resembles ADPKD, apoptosis is increased and correlates with the upregulation of caspase protein levels and activity and the downregulation of anti-apoptotic Bcl2 family proteins (Ecder et al., 2002; Tao et al., 2005b). In *cpk* mice, excessive apoptosis occurs in the interstitium while seldom evident in the cystic epithelium or noncystic tubules. The expression of various caspases, including bax and bcl-2, are also upregulated indicating that apoptotic cell death contributes to cyst formation (Ali et al., 2000). In Madin-Darby canine kidney (MDCK) cells, apoptosis was increased and overexpression of PC1 displayed resistance to thrombin/Gα12-stimulated apoptosis while PC1-silenced MDCK cells displayed enhanced thrombin-induced apoptosis (Boletta et al., 2000). In *Pkd1* mutant mice, *Pkd1* deletion in renal stromal cells by Foxd1-driven Cre showed epithelial changes and progressive cystogenesis accompanied by excessive apoptosis and proliferation (Nie and Arend, 2017). *Pkd1* conditional knockout mice also showed increased expression levels of pro-apoptotic markers and downregulation of the anti-apoptotic marker (Rogers et al., 2016). In *Pkd1*-deleted MEFs, enhanced cytochrome c release and increased apoptosis were detected which caused increased sensitivity to ROS (Zhang et al., 2015). In *Pkd2* mutant models, haploinsufficiency and loss of heterozygosity at the *Pkd2* locus results in both increased proliferation and apoptosis (Belibi et al., 2004; Wilson and Goilav, 2007; Kim et al., 2009; Wegierski et al., 2009). In addition, *Pkd2*-overexpressing transgenic mice showed typical renal cyst growth with an increase in both proliferation and apoptosis (Park et al., 2009; Zhang et al., 2015). Besides, in *Pkd1* ^*+/−*^pigs, both intrinsic and extrinsic apoptosis increased at ages of 1 month and 3 months. This provides more evidence for the correlation of apoptosis in ADPKD among different mammalian species (Wang et al., 2021).

Second, different studies have either reported no significant change or decreased apoptosis in rodent ADPKD models. In *Pkd1* ^*flox/flox*^ *:Ksp-Cre* mice, increased proliferation which correlated with increased cystic kidney, was observed, however, no significant changes in apoptosis were reported (Shibazaki et al., 2008). Meanwhile, apoptotic cells were rarely detected in kidneys of *Pkd1* knockdown mice and in kidneys from *Pkd1* ^*flox/flox*^ *:Ksp-Cre* neonates. In proximal tubule derived cell lines lacking PC1 which spontaneously formed cysts, the overall number of apoptotic nuclei in kidney tissues was very low and did not differ significantly between cystic and normal kidneys (Wei et al., 2008). In the kidneys from *Pkd1* conditional knockout and *Pkd1* hypomorphic *Pkd1* ^*nl/nl*^ mice, apoptotic cells were rarely detected (Fan et al., 2013a). In *Pkd1* ^*−/−*^ E15.5 MEKs (mouse embryonic kidneys), apoptosis was rare and negligible as detected by TUNEL assay (Zhou et al., 2013). In *Pkd1* ^*fl/fl*^ *:Smyd2* ^*+/+*^ *:Ksp-Cre* neonates, apoptosis was rare in kidneys and knockout of Smyd2 induced cyst-lining epithelial cell death in kidneys from *Pkd1* ^*fl/fl*^ *:Smyd2* ^*fl/fl*^ *:Ksp-Cre* neonates (Li et al., 2017b). The controversial role for apoptosis in PKD is due to confounding factors including: 1) the use of variable animal models versus human PKD, 2) comparison at various stages of the disease, early versus late, 3) comparison between cyst lining epithelium versus normal appearing tubules. It is evident that both cell apoptosis and proliferation are dysregulated in ADPKD, and both may contribute to the general mechanism for cyst growth. Moreover, PKD kidneys are disproportionately enlarged. If apoptosis were therefore a predominant factor in the regulation of cyst growth, one would expect the kidneys to eventually involute. This therefore suggests that apoptosis is not the primary factor of cystogenesis (Zhou and Li, 2015).

#### 4.2.2 Autophagy in ADPKD

Like the role of apoptosis in ADPKD, the role of autophagy in ADPKD is also controversial. The disruption of autophagy has been identified in diverse ADPKD animal models. However, both increased and decreased autophagy are reported. In *cpk* mice and *Han:SPRD* rats, autophagy is increased as autophagosomes were found by electron microscopy in the tubular cells lining the cysts, and enhanced autophagy components LC3-II and beclin-1 were also observed (Belibi et al., 2011). In PC1 deficient cells, basal autophagy was enhanced (Decuypere et al., 2021). However, in *Pkd1* mutant mice, the expression level of autophagy genes was decreased although LC3-II protein level showed no change (Belibi et al., 2011; Chou et al., 2018). mTOR pathway is hyperactivated and suppressed autophagic flux is detected in the heart and kidneys of *Pkd1* mutant mouse models (Atwood et al., 2020a; Atwood et al., 2020b). In the *Pkd1* mutant zebrafish model, the mTOR pathway is abnormally activated and autophagy is inhibited (Zhu et al., 2017). In *Pkd1* ^*flox/-*^ *:Ksp-Cre* mice, autophagy-related protein ULK1 and the ratio of LC3 II/LC3 I decreased, indicating autophagy is inhibited (Liu et al., 2020). In *Pkd1 transgenic* mice, the expression levels of autophagy-related genes, including Atg5, Atg12, Ulk1, Beclin-1, and Sqstm1 (p62) were down-regulated. However, the p62 protein level in cystic lining cells was increased, indicating impaired degradation of the protein by the autophagy-lysosome pathway (Chou et al., 2018). In human ADPKD patient samples, autophagy did not change though LC3 was highly increased in cystic ADPKD patient kidneys. Meanwhile, the enhanced LC3 expression in PKD mouse models enlarged renal cysts and enhanced renal failure (Lee et al., 2020).

Dysregulation of the autophagy pathway has been associated with disease progression in ADPKD, however, the mechanisms involved in the PC1- and PC2-mediated regulation of autophagy remains unclear. Treatment with hyperosmolar concentrations of sorbitol or mannitol induces PC2 dependent autophagy and downregulation of PC2 prevents inhibition of hyperosmotic stress-induced mTOR pathway activation in HeLa and HCT116 cell lines (Pena-Oyarzun et al., 2017). PC2 can bind to BECN1, a component of PI3KC3 complex and is essential to initiate autophagy, through CC1 domain, which is in the carboxy-terminal tail of PC2. Note that the PC2-BECN1 complex is required for the induction of autophagy (Pena-Oyarzun et al., 2021). In *Pkd2* knockout mice, the activation of autophagic flux was suppressed and overexpression of PC2 could increase autophagic flux (Criollo et al., 2018). While PC2 seems to be essential for stress-induced autophagy, PC1 also functions in the process of autophagy. Dysfunction of PC1 and/or PC2 in cell lines lead to reduced intracellular Ca^2+^ signaling, increased mTOR activity, increased cAMP levels and increased proliferation (Seeger-Nukpezah et al., 2015). PKD mutant cells can be more resistant against nutrient stress and delay cell death by maintaining autophagy modulated through PC1 in a PC2-dependent manner. In *PC1* knockout mice, starvation-induced autophagic response is enhanced. Although knockdown of PC2 in *PC1* knockout cells did not significantly alter autophagy level, *PC2* knockout cells showed reduced protein level of PC1 and the PC1 band seemed to be at a lower molecular weight, suggesting that the stability of PC1 protein during starvation is dependent on PC2 expression (Decuypere et al., 2021).

#### 4.2.3 Ferroptosis in ADPKD

The dysregulation of ferroptosis was detected in animal models of ADPKD and human ADPKD. The expression of antioxidant enzymes GPX and SOD and their activity were decreased in *cpk* mice and *Han:SPRD* rats (Maser et al., 2002) while lipid peroxidation is increased in human ADPKD patients (Schreiber et al., 2019), suggesting that ferroptosis plays an important role in ADPKD. Lipid peroxidation correlated with cyst growth and the activation of TMEM16A (anoctamin 1), a chloride bicarbonate transmembrane channel, inducing growth of renal cysts by lipid peroxidation while the direct inhibition of TMEM16A or inhibition of lipid peroxidation delayed cyst development in *Pkd1* mutant mice (Schreiber et al., 2019). Treatment with two potent inhibitors of TMEM16A, niclosamide and benzbromarone, significantly reduced renal cyst size in *Pkd1* ^*−/−*^ mice compared to control mice (Cabrita et al., 2020). Recently, we reported the decreased expression of the negative regulator of ferroptosis GPX4, and the increased expression of iron importers TfR1 and DMT1 in *Pkd1* mutant mouse models. This alteration in expression levels resulted in high iron levels, low GSH and GPX4 activity, increased lipid peroxidation, and a propensity for ferroptosis. 4HNE (4-hydroxynonnenal), a lipid peroxidation product, is also increased in *Pkd1* null cells and is reportedly responsible for the promotion of proliferation of *Pkd1* mutant cells through the activation of Akt, S6, Stat3, and Rb (Zhang and Li, 2022). These findings indicated that ferroptosis is highly associated with ADPKD progression.

### 4.3 Therapeutic Regulation and Implication of Regulated Cell Death in ADPKD

#### 4.3.1 Apoptosis-Based Therapeutic Strategies

In ADPKD, apoptosis may function as a double-edged sword. While apoptosis occurs in the normal-appearing, non-cystic tubules, loss of renal tissue may result in the functional progressive deterioration of kidney (Woo, 1995), increased apoptosis in ADPKD may inhibit hyperproliferation thus prevent the progression of PKD.

**FIGURE 5:**
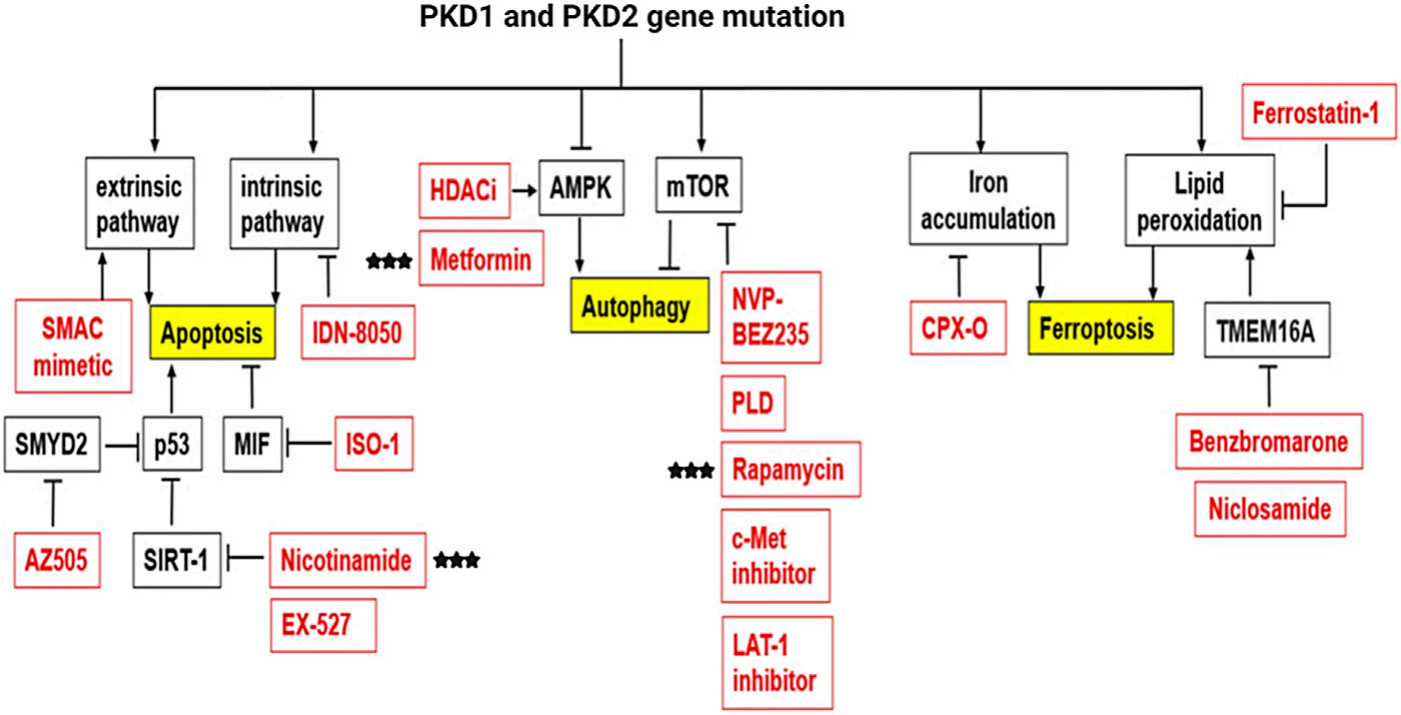
Cell death pathway and the drug targets in ADPKD. In ADPKD, mutation of PKD1/PKD2 causes dysregulation of cell death, including apoptosis, autophagy and ferroptosis. Apoptosis is regulated through extrinsic pathway and intrinsic pathway. Treatment with SMAC mimetic activates apoptosis through an extrinsic dependent pathway to delay cyst growth, while treatment with pan-caspases inhibitor IDN-8050 inhibits apoptosis through an intrinsic pathway which also reduces cyst size. Treatment with SIRT-1 inhibitors, including nicotinamide and EX-527, and Smyd2 inhibitor, AZ505, as well as MIF inhibitor, ISO-1, induced apoptosis in a p53 dependent manner and delay cyst growth. Autophagy is regulated through PKD associated AMPK and mTOR pathways. Treatment with inhibitors of mTOR pathway, including NVP-BEZ236, PLD and rapamycin as well as c-Met and LAT-1 inhibitors, delays cyst growth, and treatment with AMPK inducers, metformin and HDACi, also slows cyst growth. Ferroptosis is an iron dependent cell death characterized with the increase of lipid peroxidation. Treatment with CPX-O, a chelator of iron, inhibited cystogenesis. Treatment with ferroptosis inhibitor, Ferrostatin-1, delays cyst growth and cystic cell proliferation. TMEM16A can induce lipid peroxidation, and treatment with TMEM16A inhibitor, niclosamide and benzbromarone, significantly reduced renal cyst size in ADPKD animals (Three black stars indicate drugs in clinical trials).

Targeting apoptosis to attenuate cyst growth is quite complex (Figure 5). First, the increase in apoptosis in ADPKD suggests that inhibition of apoptosis should delay cyst progression. Short-term treatment with the pan-caspase inhibitor IDN-8050 resulted in the inhibition of caspase-3 and caspase-7 activity in kidneys of *Cy/Cy* rats (Tao et al., 2005b) and decrease of apoptosis. This inhibited renal enlargement and cystogenesis and attenuated the loss of kidney function (Tao et al., 2005a). Deletion of caspase-3 resulted in the increased caspase-7 and decreased anti-apoptotic protein Bcl-2 levels, and prolonged the survival of *cpk* mice (Tao et al., 2008). In *Han:SPRD* rats, treatment with 2-hydroxyestradiol (2-OHE) reduced apoptosis and resulted in decreased cyst growth and preservation of kidney (Anderson et al., 2012). Furthermore, in two ARPKD mouse models (*jck* juvenile cystic kidney mice and *cpk* mice), treatment with cyclin-dependent kinase (CDK) inhibitor roscovitine resulted in long-lasting arrest of cystogenesis and decreased apoptosis. The decreased expression of pro-apoptotic protein Apaf1 and caspase-2, and increased expression of Bcl-2 and Bcl-xL were also detected. The may be caused by the inhibition of Cdk5, which is responsible for anti-apoptotic effects in neurodegenerative diseases, thereby resulting in effective reduction of apoptosis in cystic kidneys (Di Giovanni et al., 2005; Bukanov et al., 2006). The Glc-Cer synthase inhibitor, Genz-123346, also slowed cyst growth by decreasing the proliferation and apoptosis in *jck* mice (Natoli et al., 2010).

Second, there is evidence to support that induction of apoptosis contributes to delayed cyst growth in ADPKD. As described above, TNF-α is an apoptosis ligand and accumulates at elevated levels in ADPKD cyst fluid (Li et al., 2008). While treatment with TNF-α alone did not induce apoptosis in either *Pkd1* wild-type or null MEK cells even at high concentration (due to the activation of NF-κB which inhibited the activity of caspase 8) (Fan et al., 2013a), treatment with TNF-α combined with the SMAC (second mitochondria-derived activator of caspases) mimetic induced apoptosis in primary cultures of mural epithelial cells from human ADPKD cysts and *Pkd1* null MEK cells, suggesting that SMAC-mimetic induces TNF-α–dependent cell death (Fan et al., 2013a). Importantly, treatment with SMAC-mimetic reduced cyst growth in *Pkd1* mutant mice (Fan et al., 2013a). After this fundamental study, subsequent studies provided more evidence to support a beneficial role for the induction of cystic renal epithelial cell apoptosis. Treatment with SMAC-mimetic also showed cyst reduction in a drosophila model of ADPKD (Millet-Boureima et al., 2019). In *Pkd1* mutant model, the deacetylase sirtuin 1 (SIRT1) is upregulated and treatment with pan-sirtuin inhibitor (nicotinamide) or a SIRT1-specific inhibitor (EX-527) delayed cyst growth through the deacetylation of p53, which released its inhibition on apoptosis in *Pkd1* mutant mice (Zhou et al., 2013). A randomized double blinded small clinic trial with the enrollment of 36 patients was conducted to determine the effect of nicotinamide in ADPKD patients. Although nicotinamide is safe and well-tolerated in ADPKD patients, there was no beneficial effect in the inhibitor treated group compared to placebo treated group (El Ters et al., 2020). SMYD2, a lysine methyltransferase, also upregulated in *Pkd1* mutant renal epithelial cells and kidneys, was shown to inhibit p53-dependent cystic renal epithelial cell apoptosis through the methylation of p53. Treatment with specific SMYD2 inhibitor AZ505 delayed cyst growth in *Pkd1* mutant kidneys by increasing p53 mediated apoptosis (Li et al., 2017b). Macrophage migration inhibitory factor (MIF) reported to be upregulated in cyst-lining epithelial cells of *Pkd1* mutant mouse kidneys, accumulates in cyst fluid of human ADPKD kidneys. Treatment with ISO-1, a MIF inhibitor, efficiently slowed cyst growth, accompanied with an increase in apoptosis (Chen et al., 2015). Genetic ablation of apoptosis regulatory genes also contributes to attenuation of cyst growth in ADPKD. Homozygous deletion of ILK (integrin-linked kinase) gene, whose product is a scaffold protein associated with multiple cellular functions including cell proliferation, resulted in increased caspase-3-mediated apoptosis and reduced cyst size of *Pkd1^fl/fl^;Pkhd1-Cre* mice (Raman et al., 2017). In *cpk* mice, haploinsufficiency of Pax2 attenuated progressing cyst growth via increased p53 mediated apoptosis. *Pax2* is known to suppress p53 transcription by binding to cis-acting regulatory sequences (Stuart et al., 1995). Apoptosis in *Pax2* heterozygous kidneys of *cpk* mice may be regulated in part by increased levels of p53 (Ostrom et al., 2000). MicroRNAs (miRNAs are short noncoding RNAs that acts as sequence-specific inhibitors of gene expression (O’Brien et al., 2018 #95). miR-21 is upregulated in renal cysts through cAMP signaling pathway and represses proapoptotic genes to inhibit cystic cell apoptosis and promotes PKD progression. Deletion of miR-21 showed attenuation of cyst growth and induction of apoptosis in kidneys from an orthologous model of ADPKD (Lakhia et al., 2016). These studies indicated a beneficial role for the induction of apoptosis in ADPKD treatment.

#### 4.3.2 Autophagy-Based Therapeutic Strategies

Direct targeting of dysfunctional autophagy in PKD animal models has been determined effective in slowing cyst growth. In *Pkd1* mutant zebrafish, knocking down the core autophagy protein Atg5 caused inhibition of autophagy and promoted cystogenesis, while treatment with a specific inducer of Beclin-1 peptide activated autophagy and also slowed cyst growth (Zhu et al., 2017). However, oral supplement with trehalose, a natural autophagy enhancer, showed no effect on the progression of ADPKD in *Pkd1* miRNA transgenic mice and could not restore impaired autophagy, suggesting that an oral supplement of trehalose may not affect the progression of ADPKD (Chou et al., 2018).

mTOR pathway plays a significant role in the regulation of autophagy and targeting mTOR pathway has been indicated effective for ADPKD (Zafar et al., 2010; Li et al., 2017a). Rapamycin has been used as an autophagy inducer that functions through inhibition of mTOR pathway. The effect of rapamycin in suppressing the aberrant epithelial proliferation of ADPKD kidney has been identified in rodent ADPKD animal models. Moreover, rapamycin was used in human studies, and while there was a reduction in TKV (total kidney volume) in rapamycin treated groups compared to placebo group, the decrease in TKV was not significant (Kim and Edelstein, 2012; Braun et al., 2014). Phospholipase D (PLD) is an enzyme of the phospholipase superfamily and its product phosphatidic acid (PA) regulate mTOR activity. The activity of PLD was elevated in PKD cells and targeting PLD with small molecule inhibitors could reduce cell proliferation and enhance the sensitivity of PKD cells to rapamycin which indicated that combining PLD inhibitors and rapamycin synergistically inhibited PKD cell proliferation (Liu et al., 2013). Two large randomized clinical trials with rapamycin in ADPKD were undertaken. However, the effect of rapamycin were unimpressive and the treatment presented an increased side-effect profile, which might be associated with the short-term administration of rapamycin and the lack of randomization (Kim and Edelstein, 2012). Other drugs targeting mTOR pathway such as NVP-BEZ235, a mTOR inhibitor, also inhibited proliferation and normalized kidney morphology in ADPKD (Liu et al., 2018). Defective ubiquitination of c-Met caused hyperactivation of mTOR in PKD and treatment with a c-Met inhibitor resulted in the inhibition of mTOR activity and blocked cystogenesis in Pkd1-null mice model of ADPKD (Qin et al., 2010). Branched-chain amino acids (BCAAs), including leucine, is an activator of mTOR pathway. Treatment with BCAA in *Pkd1* conditional knockout mice accelerated disease progression and upregulation of mTOR pathway. L-type amino acid transporter 1 (LAT-1) is a transporter of neutral amino acids, including BCAAs. Treatment with LAT-1 inhibitor reduces mTOR signaling and attenuation of cyst growth indicated that LAT-1 inhibitor is a potential therapeutic agent for ADPKD (Yamamoto et al., 2017).

Targeting AMPK pathway is also involved in the treatment of ADPKD. Metformin is an AMPK inducer and treatment with metformin slowed cyst formation in both *in vitro* and *ex vivo* models of renal cystogenesis (Takiar et al., 2011; Chang et al., 2017). In a clinical trial, it was also determined that treatment with metformin improved disease progression. In this study, it was found that metformin effects on ADPKD progression are mediated through the activation of the AMPK pathway (Seliger et al., 2018). In addition, treatment with inhibitors of histone deacetylases (HDACi) delayed cyst growth in *Pkd1* ^*−/−*^ models, partially by activating AMPK pathway (Sun et al., 2019). Furthermore, inhibition of miR-25-3p enhanced autophagy by increasing ULK1 expression and the ratio of LC3 II/LC3 I in kidneys from *Pkd1* ^*flox/−;*^ *Ksp-Cre* mice (Liu et al., 2020). These studies further support that targeting specific autophagy regulators may attenuate cyst growth, as such, they provided novel targets for the treatment of ADPKD (Figure 5).

#### 4.3.3 Ferroptosis-Based Therapeutic Strategies

Lipid peroxidation correlated with cyst growth and the activation of TMEM16A (anoctamin 1), a chloride bicarbonate transmembrane channel, inducing growth of renal cysts by lipid peroxidation while the direct inhibition of TMEM16A or inhibition of lipid peroxidation delayed cyst development in *Pkd1* mutant mice (Schreiber et al., 2019). Treatment with two potent inhibitors of TMEM16A, niclosamide and benzbromarone, significantly reduced renal cyst size in *Pkd1* ^*−/−*^ mice compared to control mice (Cabrita et al., 2020). Ferrostatin-1 is a synthetic antioxidant and also works as a lipid peroxidation inhibitor but much more efficiently. Treatment with Ferrostatin-1 inhibits ferroptosis and the proliferation of *Pkd1* mutant renal epithelial cells and kidneys (Zhang et al., 2021c). Ciclopirox (CPX) or its olamine salt (CPX-O) inhibits the activity of iron-dependent enzymes through chelation of iron. Treatment with CPX-O in PKD mice inhibited cystogenesis as seen by the decrease of cyst index and cystic cell proliferation, and the improvement of renal function, suggesting that CPX-O may delay cyst growth through affecting ferroptosis process in *Pkd1* mutant mice (Radadiya et al., 2021). In conclusion, targeting ferroptosis may be a new strategy for ADPKD treatment but still need clinical trials to identify (Figure 5).

In summary, the regulated cell death, including apoptosis, autophagy and ferroptosis, has been investigated in ADPKD, and there is evidence to suggest their involvement in cyst progression (Figure 5). In-spite of these studies, the role of regulated cell death in regulating cystogenesis in ADPKD remains unclear. Moreover, the controversial reports add to the complexity of programmed cell death in general, and how it associates to ADPKD. A major unresolved concern in the field is whether induction of regulated cell death promotes or delays cyst growth. Thus far, the answer to this question is based on two confounding factors including: 1) the type of cell death induced, and 2) the stage of the disease. While targeting regulated cell death appears to be an attractive approach for ADPKD therapy, we believe that further investigation is required to help identify more specific targets for the development of more precise therapy for ADPKD.

## 5 Conclusion and Perspective

In the last decade, an understanding of common mechanisms involved in ADPKD disease, including epigenetic modifications, inflammation, and regulated cell death, have pointed to alternative approaches for potential therapeutic intervention, with promising results emerging from multiple pre-clinical studies. The elucidation of these different mechanisms has uncovered key concerns regarding disease progression. First, the disease model and experimental techniques used, and second, the stage of the disease during which samples are collected, determine the conclusions drawn and how the data obtained is extrapolated for therapeutic purposes. Third, there exits feedback mechanisms and cross-talks between dysregulated pathways in ADPKD which create a more complex network. For example, epigenetic modifications contribute to renal damage by regulating multiple cellular processes and signaling pathways including inflammation and cell death pathways, while inflammation and programmed cell death can contribute to the renal damage progression by inducing epigenetic modifications. Therefore, efforts towards creating a comprehensive picture of the epigenetic landscape and understanding how epigenetic mechanisms affect inflammation and regulated cell death pathways should lay out the foundation for future drug development against ADPKD.

The treatment of ADPKD is a long-term effort, owing to the progressive nature of the disease. Therefore, it is important and necessary to find more selective and less toxic drugs that will be effective for long-term treatment. As such, a better understanding of the mechanisms involved in ADPKD disease progression is essential. To achieve this goal, we should develop and apply the techniques of single cell epigenomics, including single-cell DNA methylome sequencing, single-cell assay for transposase-accessible chromatin with sequencing (scATAC-seq), single-cell ChIP-sequencing and single-cell Hi-C, in our future research to better understand how epigenetic mechanisms are involved in ADPKD progression. In addition, CRISPR–Cas9 screens may be applied to identify transcriptional or epigenetic factors that modulate the immune sensitivity of cystic cells or immune activity of immune cells. A better understanding of immunosuppression mechanisms in cystic kidneys should also facilitate the identification of novel immunotherapeutic targets and the development of an immunotherapeutic strategy for ADPKD treatment. Furthermore, to establish a connection of epigenetic mechanisms and renal inflammation with regulated cell death in ADPKD and to investigate the roles of other types of regulated cell death, such as pyroptosis (a highly inflammatory cell death usually caused by microbial infection), necroptosis (a regulated inflammatory mode of cell death that mimics features of both apoptotic and necrotic cell death) and oxeiptosis (a ROS-induced caspase-independent apoptosis-like cell death) in ADPKD should facilitate the identification of more specific targets for a more precise therapy for ADPKD. We believe that an effective and long-term treatment of ADPKD can be achieved by simultaneously or serially targeting different signaling pathways.
